# Supplementary material for: Analytical Determination of the Nucleation-Prone, Low-Density Fraction of Subcooled Water
Source: Entropy (Basel). 2020 Aug 25;22(9):933. doi: 10.3390/e22090933 (PMC7597191; doi:10.3390/e22090933)
Supplement: Supplementary file 1 [file entropy-22-00933-s001.pdf]

Article

Supplementary Material to "Analytical Determination of the Nucleation-Prone, Low-Density Fraction of Subcooled Water"

Olaf Hellmuth [1,*] and Rainer Feistel [2]

[1] Leibniz Institute for Tropospheric Research (TROPOS), Permoserstraße 15, D-04318 Leipzig, Germany
[2] Leibniz Institute for Baltic Research (IOW), Seestraße 15, D-18119 Rostock-Warnemünde, Germany; rainer.feistel@io-warnemuende.de
* Correspondence: olaf.hellmuth@tropos.de

Version August 25, 2020 submitted to Journal Not Specified

Contents

SM-1 The Thermodynamic Equation Of Seawater 2010 (TEOS-10)
SM-2 On the Relevance of the Two-State Character of Subcooled Water for Ice Crystallization
SM-3 IAPWS G12-15 Formulation for the Properties of Subcooled Water
SM-3.1 Equation of State
SM-3.2 Thermodynamic Properties
SM-3.3 Validity Range
SM-3.4 Constraints on the Numerical Solution of Equation (SM-3.8)
SM-3.5 IAPWS G12-15 Thermodynamic Reference Values for the Check of the Correct Computer Implementation
SM-4 Analytical Solution of a Cubic Equation
SM-5 Table Values of the Computer-Program Verification
SM-6 Table Values of the Deviations of the Analytical from the IAPWS G12-15 [1] Reference Formulation
SM-7 Table Values of the Deviation of the Analytically from the Numerically Determined Thermodynamic Properties of Subcooled Water
SM-7.1 Mass Density
SM-7.2 Thermal Expansivity
SM-7.3 Compressibility
SM-7.4 Isobaric Heat Capacity
SM-7.5 Sound Speed
SM-7.6 Gibbs Energy and Entropy
References

SM-1 The Thermodynamic Equation Of Seawater 2010 (TEOS-10)

TEOS-10 is based on four independent thermodynamic potentials, defined as functions of the independent observables temperature, pressure, dry-air mass fraction, density, and salinity: (i) a Helmholtz function of fluid water (Wagner and Pruß [2], IAPWS R6-95 [3], known as "IAPWS-95"), (ii)

a Gibbs function of hexagonal ice (Feistel and Wagner [4], IAPWS R10-06 [5]), (iii) a Gibbs function of seasalt dissolved in water (Feistel [67], IAPWS R13-08 [8]), and (iv) a Helmholtz function for dry air (Lemmon *et al.* [9]). In combination with air–water cross-virial coefficients (Hyland and Wexler [10], Harvey and Huang [11], Feistel *et al.* [12]) this set of thermodynamic potentials is used as the primary standard for pure water (liquid, vapor, and solid), seawater and humid air from which all other properties are derived by mathematical operations, i.e., without the need for additional empirical functions.

The IAPWS-95 fluid-water formulation is based on the evaluation of a comprehensive and consistent data set which was assembled from a total of about 20,000 experimental data of water. Wagner and Pruß [2] took into account all available information given in the scientific articles which described the data collection underlying the development of the thermal equation of state of water. The authors critically reexamined the available data sets with respect to their internal consistency and their basic applicability for the development of a new equation of state for water. Only those data were incorporated into the final nonlinear fitting procedure, which were judged to be of high quality. These selected data sets took into account experimental data which were available by the middle of the year 1994 (Wagner and Pruß [2]). The availability of reliable experimental data on subcooled liquid water (i.e., metastable with respect to the solid form of water) was restricted to a few data sets for several properties only along the isobar p=1013.25 hPa, which set the lower limit of the temperature range for the validity of IAPWS-95 for liquid water (and so of TEOS-10) to T=236 K. This temperature is called the temperature of homogeneous ice nucleation (or homogeneous freezing temperature) at p=1013.25 hPa and represents the lower limit below which it is very difficult to subcool water at this pressure. The assessment of the accuracy of the IAPWS-95 formulation in the temperature range of subcooled liquid water (Wagner and Pruß [2], Section 7.3.2 therein) revealed that TEOS-10 fully satisfies the meteorological needs with respect to accuracy down to this temperature. The IAPWS-95 fluid-water formulation is valid in the entire *stable* fluid region of H_2O from the melting-pressure curve to 1273 K at pressures up to 1000 MPa; the lowest temperature on the melting-pressure curve is 251.165 K (at 208.566 MPa) (IAPWS R6-95 [3], Section 5 therein). In the stable fluid region, the formulation can also be extrapolated beyond these limits. Based on a comparison with experimental data, the formulation was reported to behave reasonably when extrapolated into the metastable regions, i.e., for superheated liquid at positive pressures (liquid-gas metastable region), and for subcooled liquid (solid-liquid metastable region). The available data were reported to be in fair agreement with the Helmholtz free energy formulation, but some recent high-pressure data are not represented within their uncertainties (IAPWS R6-95 [3], Section 5 therein).

SM-2 On the Relevance of the Two-State Character of Subcooled Water for Ice Crystallization

The two-state character of subcooled water has implications for ice crystallization which arise from an empirical judgement of Ostwald [13] (pp. 306–309 therein), according to which *"in the course of transformation of an unstable (or metastable) state into a stable one the system does not go directly to the most stable conformation (corresponding to the modification with the lowest free energy) but prefers to reach intermediate stages (corresponding to other possible metastable modifications) having the closest free energy difference to the initial state"* (e.g., Schmelzer *et al.* [1415], Schmelzer [16], Schmelzer *et al.* [17], Gutzow and Schmelzer [18], Schmelzer and Abyzov [19]). Later, this perception has become known as Ostwald's rule of stages. However, with respect to phase transformation processes Schmelzer *et al.* [14] and Gutzow and Schmelzer [18] emphasized the existence of exceptions from this rule, leading to the conclusion that Ostwald's rule of stages cannot be considered as a generally valid thermodynamically founded law. This statement is in line with results from statistical mechanical modelling of anisotropic nanoparticle crystallization carried out by Hedges and Whitelam [20], who argued that although often upheld, the rule is without theoretical foundation and is not universally obeyed.

In advancing Ostwald's rule of stages Schmelzer *et al.* [15] (see also Schmelzer *et al.* [17], Gutzow and Schmelzer [18], Schmelzer and Abyzov [19], Schmelzer *et al.* [21]) proposed its generalization as follows: *"In phase transformation processes, the structure and properties of the critical nucleus may differ qualitatively from the properties both of the ambient and the newly evolving macrophases. Those classes of critical clusters determine the process of the transformation which correspond to a minimum of the work of critical cluster formation (as compared with all other possible alternative structures and compositions which may be formed at the given thermodynamic constraints)."* As emphasized by those authors, this generalization implies – as in Ostwald's formulation – that critical clusters with quite different compositions may be formed but does not restrict these possible cluster states to structures and compositions essentially determined by the evolving possibly stable or metastable macrophases. In Schmelzer *et al.* [15], [22], [21], [17], the generalized Ostwald's rule of stages was employed as a well-founded principle for a generalization of Gibbs' classical way of determination of the work of critical cluster formation (Gibbs [23]), known as generalized Gibbs' theory. In the latter, as reference states for the determination of the bulk contributions to the properties of the critical clusters, the authors did not choose the quantities of the respective macrophase in equilibrium with the ambient phase; instead, they used bulk contributions which correspond to the most appropriate structure and properties, i.e., to those structures and properties which result in a minimum of the work of critical cluster formation.

Upon undercooling of water at sufficiently high pressure the character of the liquid changes by transformation from a dense, high-entropic phase to a less dense, low-entropic (more ordered) phase (Debenedetti and Stanley [24]). According to Ostwald's rule of stages, the low-entropic, more ordered phase can be thought as an intermediate stage (corresponding to other possible metastable modifications) having the closest free energy difference to the initial state. Following this perception, new ice embryos are expectable to be more likely formed from the low-density water fraction B rather than from the high-density fraction A. This would imply that the thermodynamic driving force of ice crystallization (see, e.g., Hellmuth *et al.* [25]) should be scaled in terms of the difference $\Delta G^{\mathrm{B}}_{\beta\alpha} = G^{\mathrm{B}}_{\beta}(T,p) - G_{\alpha}(T,p)$ between the Gibbs energies of low-density water B (represented by $G^{\mathrm{B}}_{\beta}(T,p)$ with subscript β for subcooled water) and ice (represented by $G_{\alpha}(T,p)$ with subscript α for ice) rather than in terms of the difference $\Delta G_{\beta\alpha} = G_{\beta}(T,p,x) - G_{\alpha}(T,p)$ between mean-state water (represented by $G_{\beta}(T,p,x)$) and ice. Such perception of heterogeneity has some conceptual similarity to that employed in Abyzov *et al.* [26], who assumed an increase of the average size of the "structural units" of the maternal phase with decreasing temperature. This structural coarsening is identified with the cooperatively rearranging regions in the liquids, the increase of which is a consequence of a more complex kinetics as compared to a quasi-one-component description of the liquid commonly employed in classical nucleation theory. As a consequence, crystal nucleation in glass-forming liquids is assumed to proceed with detectable rates only in the liquid-like (soft) regions and to be suppressed in solid-like (rigid) parts of the liquid. In their model, the fraction of liquid-like and solid-like regions in dependence on temperature serves as a closure parameter, determined to achieve a comprehensive agreement between classical nucleation theory and experiment not only at relatively high temperatures but also at temperatures lower than that of the nucleation rate maximum.

An implication of these considerations is the determination of the nucleation rate as a mean value. Owing to the nonlinear dependence of the nucleation rate coefficient on temperature and pressure, the nucleation rate coefficient at mean-state conditions, J, differs from the mean-state nucleation rate coefficient, $\overline{J}$, tentatively defined by the following mixing rule:

$$\begin{aligned}
J(\Delta G_{\beta\alpha}) &\neq \overline{J} \approx \underbrace{xJ\left(\Delta G^{\mathrm{B}}_{\beta\alpha}\right)}_{\text{from structure B}} + \underbrace{(1-x)J\left(\Delta G^{\mathrm{A}}_{\beta\alpha}\right)}_{\text{from structure A}} , \\
\Delta G_{\beta\alpha} &= G_{\beta}(T,p,x) - G_{\alpha}(T,p) , \\
\Delta G^{\mathrm{A}}_{\beta\alpha} &= G^{\mathrm{A}}_{\beta}(T,p) - G_{\alpha}(T,p) , \\
\Delta G^{\mathrm{B}}_{\beta\alpha} &= G^{\mathrm{B}}_{\beta}(T,p) - G_{\alpha}(T,p) .
\end{aligned}$$

The quantification of the differences $\Delta J_1 = J(\Delta G^{\mathrm{B}}_{\beta\alpha}) - J(\Delta G_{\beta\alpha})$ and $\Delta J_2 = J(\Delta G_{\beta\alpha}) - \overline{J}$ is, however, beyond the scope of the present analysis.

118 # SM-3 IAPWS G12-15 Formulation for the Properties of Subcooled Water

119 ## SM-3.1 Equation of State

The EoS according to the IAPWS G12-15 [1] formulation (Equation (1) therein) is given by the dimensionless Gibbs energy ψ as function of temperature T and pressure p:

$$\begin{aligned}\psi(T,p) &= \frac{\widehat{g}(T,p)}{R_\mathrm{W} T_\mathrm{LL}} \\ &= \psi^\mathrm{r}(\tau,\pi) + (\tau+1)\Big[x_\mathrm{e} L(\tau,\pi) + x_\mathrm{e}\ln x_\mathrm{e} + (1-x_\mathrm{e})\ln(1-x_\mathrm{e}) + \omega(\pi) x_\mathrm{e}(1-x_\mathrm{e})\Big] \ .\end{aligned} \tag{SM-3.1}$$

Here, $\widehat{g}(T,p)$ denotes the specific Gibbs energy of subcooled water. The quantity x_e is the equilibrium mole fraction of the low-density structure of water in the two-state water mixture, and serves as a determinable parameter of the EoS. The other quantities are the temperature of the liquid-liquid critical point T_LL, the specific gas constant of water R_W, the dimensionless function of the ordering field L, the dimensionless interaction parameter ω, and the reduced temperature τ and the reduced pressure π defined as (IAPWS G12-15 [1], Equation (2) therein):

$$\tau = \frac{T}{T_\mathrm{LL}} - 1 \,, \quad \pi = \frac{p}{\widehat{\varrho}_0 R_\mathrm{W} T_\mathrm{LL}} \ . \tag{SM-3.2}$$

The quantity $\widehat{\varrho}_0$ is the reference mass density of subcooled water. The values of T_LL, $\widehat{\varrho}_0$, and R_W are given in Table SM-3.1.1. The dimensionless background Gibbs energy ψ^r reads (IAPWS G12-15 [1], Equation (3) therein):

$$\psi^\mathrm{r}(\tau,\pi) = \sum_{i=1}^{20} c_i (\tau+1)^{a_i} (\pi+\pi_0)^{b_i} \exp\left[-d_i(\pi+\pi_0)\right] \ . \tag{SM-3.3}$$

The reference dimensionless pressure π_0 is defined by

$$\pi_0 = \frac{p_0}{\widehat{\varrho}_0 R_\mathrm{W} T_\mathrm{LL}} \,, \tag{SM-3.4}$$

with p_0 given in Table SM-3.1.1. The coefficients a_i, b_i, and c_i are presented in Table SM-3.1.2. The order field $L(\tau,\pi)$ is defined by the following relations (IAPWS G12-15 [1], Equations (4) and (5) therein):

$$\begin{aligned} L(\tau,\pi) &= L_0 \left(\frac{K_2}{2k_1k_2}\right)\left[1 + k_0k_2 + k_1(\pi + k_2\tau) - K_1(\tau,\pi)\right] \,, \\ K_1(\tau,\pi) &= \sqrt{\left[1 + k_0k_2 + k_1(\pi - k_2\tau)\right]^2 - 4k_0k_1k_2(\pi - k_2\tau)} \,, \\ K_2 &= \sqrt{1 + k_2^2} \ . \end{aligned} \tag{SM-3.5}$$

The values of L_0, k_0, k_1, and k_2 are given in Table SM-3.1.1. The quantity ω denotes the interaction parameter, which depends linearly on pressure (IAPWS G12-15 [1], Equation (6) therein):

$$\omega(\pi) = 2 + \omega_0\pi \ . \tag{SM-3.6}$$

The reference parameter ω_0 is given in Table SM-3.1.1. The equilibrium mole fraction of the low-density structure of water is a function of temperature and pressure, x_e=$x_\mathrm{e}(T,p)$, which is determined by minimizing the Gibbs energy in dependence on x_e at isothermal and isobaric conditions (IAPWS G12-15 [1], Equation (7) therein):

$$\left(\frac{\partial\psi(T,p,x)}{\partial x}\right)_{T,p} = 0 \ . \tag{SM-3.7}$$

Table SM-3.1.1. Parameter values for the equation of state given by Equations (SM-3.1)–(SM-3.8). Taken from IAPWS G12-15 [1] (Table 1 therein).

Parameter	Value	Parameter	Value	Unit
ω_0	0.521 226 9	p_0	300×10^6	Pa
L_0	0.763 179 54	T_{LL}	228.2	K
k_0	0.072 158 686	$\hat{\varrho}_0$	1081.648 2	$\mathrm{kg\,m^{-3}}$
k_1	−0.315 692 32	R_W	461.523 087	$\mathrm{J\,kg^{-1}\,K^{-1}}$
k_2	5.299 260 8			

Table SM-3.1.2. Parameter values for the dimensionless regular background Gibbs energy ψ^r in Equation (SM-3.3). Taken from IAPWS G12-15 [1] (Table 2 therein).

i	c_i	a_i	b_i	d_i
1	$-8.157\,068\,138\,165\,5\times10^{0}$	0	0	0
2	$1.287\,503\,2\times10^{0}$	0	1	0
3	$7.090\,167\,359\,801\,2\times10^{0}$	1	0	0
4	$-3.277\,916\,1\times10^{-2}$	−0.2555	2.1051	−0.0016
5	$7.370\,394\,9\times10^{-1}$	1.5762	1.1422	0.6894
6	$-2.162\,862\,2\times10^{-1}$	1.6400	0.9510	0.0130
7	$-5.178\,247\,9\times10^{0}$	3.6385	0	0.0002
8	$4.229\,351\,7\times10^{-4}$	−0.3828	3.6402	0.0435
9	$2.359\,210\,9\times10^{-2}$	1.6219	2.0760	0.0500
10	$4.377\,375\,4\times10^{0}$	4.3287	−0.0016	0.0004
11	$-2.996\,777\,0\times10^{-3}$	3.4763	2.2769	0.0528
12	$-9.655\,801\,8\times10^{-1}$	5.1556	0.0008	0.0147
13	$3.759\,528\,6\times10^{0}$	−0.3593	0.3706	0.8584
14	$1.263\,244\,1\times10^{0}$	5.0361	−0.3975	0.9924
15	$2.854\,269\,7\times10^{-1}$	2.9786	2.9730	1.0041
16	$-8.599\,494\,7\times10^{-1}$	6.2373	−0.3180	1.0961
17	$-3.291\,615\,3\times10^{-1}$	4.0460	2.9805	1.0228
18	$9.001\,961\,6\times10^{-2}$	5.3558	2.9265	1.0303
19	$8.114\,972\,6\times10^{-2}$	9.0157	0.4456	1.6180
20	$-3.278\,821\,3\times10^{0}$	1.2194	0.1298	0.5213

Evaluation of Equation (SM-3.7) with $\psi(T,p,x_e)$ from Equation (SM-3.1) leads to the following transcendental equation $\mathcal{F}(x_e)=0$, the numerical root of which yields the sought-after value of x_e (IAPWS G12-15 [1], Equation (8) therein):

$$\mathcal{F}(x_e) = L(\tau,\pi) + \ln\left(\frac{x_e}{1-x_e}\right) + \omega(\pi)(1-2x_e) = 0\,. \tag{SM-3.8}$$

SM-3.2 Thermodynamic Properties

According to IAPWS G12-15 [1] (Equations (12)–(17) and Table 3 therein) the thermodynamic properties can be derived from the derivatives of $\psi(T,p)$ at the equilibrium mole fraction x_e. As a matter of convenience the corresponding dependencies are expressed in terms of the order parameter ϕ and the susceptibility χ (IAPWS G12-15 [1], Equation (12) therein):

$$\phi(x_e) = 2x_e - 1\,, \quad \chi(\pi,x_e) = \left(\frac{2}{1-(\phi(x_e))^2} - \omega(\pi)\right)^{-1}\,. \tag{SM-3.9}$$

Table SM-3.2.1. Derivatives of $L(\tau,\pi)$ and $\psi^{\mathrm{r}}(\tau,\pi)$. To simplify the annotation of the derivatives of ψ^{r} (right column), the following shorthand definitions are used: $\overline{\tau}=\tau+1$ and $\overline{\pi}=\pi+\pi_0$. Taken from IAPWS G12-15 [1] (Table 3 therein).

Derivatives of L	Derivatives of ψ^{r}
$L_\tau = \frac{L_0K_2}{2}\left(1+\frac{1-k_0k_2+k_1(\pi-k_2\tau)}{K_1}\right)$	$\psi^{\mathrm{r}}_\tau = \sum_{i=1}^{20} c_i a_i \overline{\tau}^{a_i-1}\overline{\pi}^{b_i} e^{-d_i\overline{\pi}}$
$L_\pi = \frac{L_0K_2(K_1+k_0k_2-k_1\pi+k_1k_2\tau-1)}{2k_2K_1}$	$\psi^{\mathrm{r}}_\pi = \sum_{i=1}^{20} c_i \overline{\tau}^{a_i}\overline{\pi}^{b_i-1}(b_i-d_i\overline{\pi}) e^{-d_i\overline{\pi}}$
$L_{\tau\tau} = -\frac{2L_0K_2k_0k_1k_2^2}{K_1^3}$	$\psi^{\mathrm{r}}_{\tau\tau} = \sum_{i=1}^{20} c_i a_i(a_i-1) \overline{\tau}^{a_i-2}\overline{\pi}^{b_i} e^{-d_i\overline{\pi}}$
$L_{\tau\pi} = \frac{2L_0K_2k_0k_1k_2}{K_1^3}$	$\psi^{\mathrm{r}}_{\tau\pi} = \sum_{i=1}^{20} c_i a_i \overline{\tau}^{a_i-1}\overline{\pi}^{b_i-1}(b_i-d_i\overline{\pi}) e^{-d_i\overline{\pi}}$
$L_{\pi\pi} = -\frac{2L_0K_2k_0k_1}{K_1^3}$	$\psi^{\mathrm{r}}_{\pi\pi} = \sum_{i=1}^{20} c_i \overline{\tau}^{a_i}\overline{\pi}^{b_i-2}\left[(d_i\overline{\pi}-b_i)^2-b_i\right] e^{-d_i\overline{\pi}}$

In the following equations, the subscripts τ and π indicate partial derivatives with respect to the subscripted quantities τ and π. Expressions for the derivatives of $L(\tau,\pi)$ and $\psi^{\mathrm{r}}(\tau,\pi)$ with respect to τ and π are presented in Table SM-3.2.1. As the equilibrium value of x_{e} is defined by Equation (SM-3.7), it is considered a function of τ and π.

1. Specific volume $\widehat{v}=1/\widehat{\varrho}$ (with $\widehat{\varrho}$ denoting the mass density) (IAPWS G12-15 [1], Equation (13) therein):

$$\widehat{v}(\tau,\pi) = \frac{1}{\widehat{\varrho}_0(\tau,\pi)}\left\{\frac{\tau+1}{2}\left[\frac{\omega_0}{2}\left(1-\phi^2(x_{\mathrm{e}})\right)+L_\pi(\tau,\pi)\left(\phi(x_{\mathrm{e}})+1\right)\right] +\psi^{\mathrm{r}}_\pi(\tau,\pi)\right\}. \tag{SM-3.10}$$

2. Specific entropy $\widehat{s}$ (IAPWS G12-15 [1], Equation (14) therein):

$$\widehat{s}(\tau,\pi) = -R_{\mathrm{W}}\left\{\frac{(\tau+1)L_\tau(\tau,\pi)}{2}\left(\phi(x_{\mathrm{e}})+1\right) +\left[x_{\mathrm{e}}L(\tau,\pi)+x_{\mathrm{e}}\ln x_{\mathrm{e}}+(1-x_{\mathrm{e}})\ln(1-x_{\mathrm{e}})+\omega(\pi)x_{\mathrm{e}}(1-x_{\mathrm{e}})\right]+\psi^{\mathrm{r}}_\tau(\tau,\pi)\right\}. \tag{SM-3.11}$$

3. Isothermal compressibility, κ_T, thermal expansion coefficient, α_p, specific isobaric heat capacity, $\widehat{c}_p$ (IAPWS G12-15 [1], Equation (15) therein):

$$\kappa_T(\tau,\pi) = \frac{\widehat{\varrho}(\tau,\pi)}{\widehat{\varrho}_0^2 R_{\mathrm{W}} T_{\mathrm{LL}}}\left\{\frac{\tau+1}{2}\left[\chi\left(L_\pi(\tau,\pi)-\omega_0\phi(x_{\mathrm{e}})\right)^2 -\left(\phi(x_{\mathrm{e}})+1\right)L_{\pi\pi}(\tau,\pi)\right]-\psi^{\mathrm{r}}_{\pi\pi}(\tau,\pi)\right\},$$

$$\begin{aligned}
\alpha_p(\tau,\pi) &= \frac{\widehat{\varrho}(\tau,\pi)}{\widehat{\varrho}_0 R_\mathrm{W} T_\mathrm{LL}} \Bigg\{ \frac{L_{\tau\pi}(\tau,\pi)}{2}(\tau+1)\Big(\phi(x_\mathrm{e})+1\Big) \\
&+\frac{1}{2}\left[\frac{\omega_0\left(1-\phi^2(x_\mathrm{e})\right)}{2}+L_\pi(\tau,\pi)\Big(\phi(x_\mathrm{e})+1\Big)\right] \\
&-\frac{(\tau+1)L_\tau(\tau,\pi)}{2}\chi\Big(L_\pi(\tau,\pi)-\omega_0\phi(x_\mathrm{e})\Big)+\psi^\mathrm{r}_{\tau\pi}(\tau,\pi)\Bigg\}\,, \qquad \text{(SM-3.12)} \\
\widehat{c}_p(\tau,\pi) &= -R_\mathrm{W}(\tau+1)\Bigg\{L_\tau(\tau,\pi)\Big(\phi(x_\mathrm{e})+1\Big) \\
&+\frac{1}{2}(\tau+1)\left[L_{\tau\tau}(\tau,\pi)\Big(\phi(x_\mathrm{e})+1\Big)-(L_\tau(\tau,\pi))^2\chi\right]+\psi^\mathrm{r}_{\tau\tau}(\tau,\pi)\Bigg\}\,.
\end{aligned}$$

4. Specific isochoric heat capacity, $\widehat{c}_v$ (IAPWS G12-15 [1], Equation (16) therein):

$$\widehat{c}_v(\tau,\pi)=\widehat{c}_p(\tau,\pi)-\frac{T(\alpha_p(\tau,\pi))^2}{\widehat{\varrho}(\tau,\pi)\kappa_T(\tau,\pi)}\,. \qquad \text{(SM-3.13)}$$

5. Speed of sound, w (IAPWS G12-15 [1], Equation (17) therein):

$$\begin{aligned}
w(\tau,\pi) &= \left[\widehat{\varrho}(\tau,\pi)\kappa_T(\tau,\pi)\frac{\widehat{c}_v(\tau,\pi)}{\widehat{c}_p(\tau,\pi)}\right]^{-1/2} \\
&= \left[\widehat{\varrho}(\tau,\pi)\kappa_T(\tau,\pi)-\frac{T(\alpha_p(\tau,\pi))^2}{\widehat{c}_p(\tau,\pi)}\right]^{-1/2}\,. \qquad \text{(SM-3.14)}
\end{aligned}$$

SM-3.3 Validity Range

The EoS of subcooled water given by Equations (SM-3.1)–(SM-3.14) is valid for metastable liquid water from the homogeneous ice nucleation temperature, $T_\mathrm{H}(p)$, to the melting temperature, $T_\mathrm{M}(p)$, at pressures in the interval $0 \le p \le 400\,\mathrm{MPa}$:

$$T_\mathrm{H}(p) \le T \le T_\mathrm{M}(p)\,, \quad 0 \le p \le 400\,\mathrm{MPa}\,.$$

The equation remains also valid for stable liquid states up to 300 K in the same pressure range, where IAPWS-95 remains the recommended formulation, and behaves reasonably when extrapolated to 1000 MPa. The homogeneous-ice nucleation curve can be approximated by the fits given in Equations (SM-3.15) and (SM-3.16) (IAPWS G12-15 [1], Equations (18) and (19) therein):

$$\begin{aligned}
\frac{p_\mathrm{H}}{\mathrm{MPa}} &= 0.1+228.27\left(1-\theta^{6.243}\right)+15.724\left(1-\theta^{79.81}\right)\,, \\
\theta &= T/235.15\mathrm{K}\,,\ 0\,\mathrm{MPa} \le p \le 198.9\,\mathrm{MPa}\,, \qquad \text{(SM-3.15)}
\end{aligned}$$

$$\begin{aligned}
\frac{T_\mathrm{H}}{\mathrm{K}} &= 172.82+0.03718\left(\frac{p}{\mathrm{MPa}}\right)+3.403\times10^{-5}\left(\frac{p}{\mathrm{MPa}}\right)^2 \\
&-1.573\times10^{-8}\left(\frac{p}{\mathrm{MPa}}\right)^3\,,\ 198.9\,\mathrm{MPa} \le p \le 1500\,\mathrm{MPa}\,. \qquad \text{(SM-3.16)}
\end{aligned}$$

The slope of the ice-nucleation curve exhibits a discontinuity at the break point $(T_\mathrm{BP}, p_\mathrm{BP}) = (181.4\,\mathrm{K}, 198.9\,\mathrm{MPa})$. At this point both curves intersect. Equation (SM-3.15) should be used for pressures $p<p_\mathrm{BP}$, and Equation (SM-3.16) for pressures $p_\mathrm{BP}<p$.

Table SM-3.3.1. Regression coefficients a_k, n_k for $p_\text{M}(T)$, Equation (SM-3.17), and b_k for the $T_\text{M}(p)$, Equation (SM-3.18). Taken from Feistel [27].

k	$p_\text{M}(T)$, Equation (SM-3.17) a_k	n_k	$T_\text{M}(p)$, Equation (SM-3.18) b_k
1	$0.119\,539\,337 \cdot 10^{7}$	3	$-1.663\,561\,044\,845\,51 \cdot 10^{-7}$
2	$0.808\,183\,159 \cdot 10^{5}$	25.75	$-2.135\,192\,419\,794\,06 \cdot 10^{-13}$
3	$0.333\,826\,860 \cdot 10^{4}$	103.75	$3.529\,674\,053\,418\,77 \cdot 10^{-20}$
4			$-2.731\,845\,252\,362\,81 \cdot 10^{-26}$

Table SM-3.4.1. Subintervals for the numerical determination of the equilibrium mole fraction of low-density water, x_e, in dependence on $L(\tau,\pi)$ and $\omega(\pi)$. The "min" function returns the smallest value of its arguments. Taken from IAPWS G12-15 [1] (Table 4 therein).

ω interval	x_e subinterval Lower bound	Upper bound
$\omega < \frac{10}{9}[\ln(19) - L]$	0.049	0.5
$\frac{10}{9}[\ln(19) - L] \le \omega < \frac{50}{49}[\ln(99) - L]$	0.0099	0.051
$\frac{50}{49}[\ln(99) - L] \le \omega$	$0.99 \exp\left[-\frac{50L}{49} - \omega\right]$	$\min\left[1.1\exp(-L-\omega), 0.0101\right]$

To complete the specification of the validity range of the EoS, here we include also the determination of the melting curve, which is not part of the IAPWS G12-15 [1] formulation. The correlation equation for the melting pressure was developed by Wagner (2006) (cited in Feistel [27], Appendix 1 therein) on the base of the guidelines IAPWS [28] and IAPWS [29] and deviates from the melting curve less than its predecessor published in Wagner and Pruß [2] (Equation (2.16) therein):

$$\frac{p_\text{M} - p_\text{TP}}{p_\text{TP}} = \sum_{\text{k}=1}^{3} a_\text{k}\left[1 - \left(\frac{T}{T_\text{TP}}\right)^{n_\text{k}}\right], \quad T_\text{TP} = 273.16\,\text{K}, \quad p_\text{TP} = 611.657\,\text{Pa}. \tag{SM-3.17}$$

Here, T_TP and p_TP denote the temperature and pressure of the triple point. The correlation equation for the melting temperature was developed by Feistel [27] (Appendix 2 therein) also on the base of the guidelines IAPWS [28] and IAPWS [29] and deviates from the melting curve significantly less than its predecessor:

$$\frac{T_\text{M} - T_\text{TP}}{T_\text{TP}} = \sum_{\text{k}=1}^{4} b_\text{k}\left[\frac{p - p_\text{TP}}{p_\text{TP}}\right]^\text{k}. \tag{SM-3.18}$$

The regression coefficients a_k, n_k and b_k are presented in Table SM-3.3.1.

SM-3.4 Constraints on the Numerical Solution of Equation (SM-3.8)

The numerical solution of Equation (SM-3.8) delivers the equilibrium mole fraction of low-density water, which is located in the interval $0 < x_\text{e} < 1$. Depending on the values of $L(\tau,\pi)$ and $\omega(\pi)$, IAPWS G12-15 [1] (Table 4 therein) distinguished three different subintervals, defined by lower and upper limits of the sought-after equilibrium value of x_e, which are presented in Table SM-3.4.1.

SM-3.5 IAPWS G12-15 Thermodynamic Reference Values for the Check of the Correct Computer Implementation

IAPWS G12-15 [1] (Table 5 therein) provides reference values for the check of the correct implementation of the EoS calculus. These values are listed in Table SM-3.5.1. In order to reproduce

these table values to the number of given digits, the mole fraction x_e should be numerically determined with a resolution of 10^{-10}.

Table SM-3.5.1. Thermodynamic reference values for check of the correct computer implementation. Taken from IAPWS G12-15 [1] (Table 5 therein).

$\frac{T}{\mathrm{K}}$	$\frac{p}{\mathrm{MPa}}$	$\frac{\widehat{\varrho}}{\mathrm{kg\,m^{-3}}}$	$\frac{\alpha_p}{10^{-4}\mathrm{K}^{-1}}$	$\frac{\kappa_T}{10^{-4}\mathrm{MPa}^{-1}}$	$\frac{\widehat{c}_p}{\mathrm{J\,kg^{-1}\,K^{-1}}}$	$\frac{w}{\mathrm{m\,s^{-1}}}$	x	L
273.15	0.101325	999.842 29	−0.683 042	5.088 499	4 218.300 2	1 402.388 6	0.096 654 715 5	0.621 204 74
235.15	0.101325	968.099 99	−29.633 816	11.580 785	5 997.563 2	1 134.585 5	0.255 102 858 7	0.091 763 68
250	200	1090.456 77	3.267 768	3.361 311	3 708.390 2	1 668.202 0	0.030 429 266 7	0.723 770 81
200	400	1185.028 00	6.716 009	2.567 237	3 338.525 0	1 899.329 4	0.007 170 080 9	1.155 396 5
250	400	1151.715 17	4.929 927	2.277 029	3 757.214 4	2 015.878 2	0.005 358 836 6	1.434 514 5

142 SM-4 Analytical Solution of a Cubic Equation

Starting point of the analytical solution is the following general form of the cubic equation:

$$Ax^3 + Bx^2 + Cx + D = 0\,, \quad x \in \mathbb{C}; \quad A, B, C \in \mathbb{R}\,. \tag{SM-4.1}$$

Here, x denotes the sought-after root of the equation, which is element of the complex numbers. The parameters A, B, C, and D are real numbers. Equation (SM-4.1) can be solved by means of *Cardano's method*, the rationale of which can be found, e.g., in Gellert *et al.* [30] (pp. 104–207 therein). Employing the transformations

$$r = \frac{B}{A}\,, \quad s = \frac{C}{A}\,, \quad t = \frac{D}{A}\,, \quad A \neq 0\,,$$

one arrives at the normal form of the cubic equation:

$$x^3 + rx^2 + sx + t = 0\,, \quad x \in \mathbb{C}; \quad r, s, t \in \mathbb{R}\,. \tag{SM-4.2}$$

In the set of complex numbers, each cubic equation has three solutions, which can coincide. One of these solutions is real, the other two are either real too or conjugate complex. According to Cardano's formula, the application of the transformation

$$x = y - \frac{r}{3} \tag{SM-4.3}$$

to the normal form yields the reduced form, in which the quadratic term is absent:

$$y^3 + py + q = 0\,, \quad p = s - \frac{r^2}{3}\,, \quad q = \frac{2r^3}{27} - \frac{sr}{3} + t\,. \tag{SM-4.4}$$

For the sought-after solution y the following ansatz is used:

$$y = u + v\,.$$

This ansatz results in the following equation:

$$(u+v)^3 + p(u+v) + q = 0 \quad \text{or, equivalently} \quad u^3 + v^3 + q + (u+v)(3uv+p) = 0\,.$$

This is one equation for the two unknown variables u and v. To satisfy the remaining degree of freedom, a side condition is employed which removes the term $(u+v)(3uv+p)$:

$$3uv + p = 0\,.$$

Therewith, one obtains the following two equations for the determination of u and v:

$$u^3 + v^3 = -q \quad \rightsquigarrow \text{quadratization} \rightsquigarrow \quad u^6 + 2u^3v^3 + v^6 = q^2\,, \tag{SM-4.5}$$

$$uv = -\frac{p}{3} \quad \rightsquigarrow \text{four times the third power} \rightsquigarrow \quad 4u^3v^3 = -4\left(\frac{p}{3}\right)^3\,. \tag{SM-4.6}$$

Subtraction of Equation (SM-4.6) from Equation (SM-4.5) yields:

$$(u^3 - v^3)^2 = q^2 + 4\left(\frac{p}{3}\right)^3 = 4\underbrace{\left[\left(\frac{q}{2}\right)^2 + \left(\frac{p}{3}\right)^3\right]}_{=\Delta} \quad \rightsquigarrow \quad u^3 - v^3 = \pm 2\sqrt{\Delta}\,. \tag{SM-4.7}$$

Combining the first form in Equation (SM-4.5) with the second form in Equation (SM-4.7),

$$u^3 + v^3 = -q\,, \quad u^3 - v^3 = \pm 2\sqrt{\Delta}\,,$$

one arrives at the following solutions for u and v:

$$u^3 = -\frac{q}{2} \pm \sqrt{\Delta}\,, \quad v^3 = -\frac{q}{2} \mp \sqrt{\Delta}\,. \tag{SM-4.8}$$

Upon interchanging the upper signs in front of the roots with the lower ones the quantity u^3 migrates to v^3, while the equations $u^3 + v^3 + q = 0$ and $uv = -p/3$ remain unchanged. Therefore it is sufficient, to consider only one pair of signs, e.g., the upper one.

Each third root of a complex number has three values; next to x_1 there are two further solutions x_2 and x_3 with

$$x_2 = \varepsilon_2 x_1\,, \quad x_3 = \varepsilon_3 x_1\,, \quad \varepsilon_2 = \frac{1}{2}\left(-1 + i\sqrt{3}\right)\,, \quad \varepsilon_3 = \frac{1}{2}\left(-1 - i\sqrt{3}\right)\,.$$

Therewith, one obtains the following solutions for u and v:

$$\begin{aligned} u_1 &= \sqrt[3]{-\frac{q}{2} + \sqrt{\Delta}}\,, \quad u_2 = \varepsilon_2 u_1\,, \quad u_3 = \varepsilon_3 u_1\,, \\ v_1 &= \sqrt[3]{-\frac{q}{2} - \sqrt{\Delta}}\,, \quad v_2 = \varepsilon_2 v_1\,, \quad v_3 = \varepsilon_3 v_1\,. \end{aligned} \tag{SM-4.9}$$

For $y = u_i + v_j$ with $i = 1,2,3$ and $j = 1,2,3$ one would obtain nine solutions of the cubic equation. However, the number of solutions reduces to the following three only,

$$y_1 = u_1 + v_1\,, \quad y_2 = u_2 + v_3\,, \quad y_3 = u_3 + v_2\,,$$

because the side constraint $u_i v_j = -p/3$ is fulfilled only for $u_1 v_1$, $u_2 v_3$ and $u_3 v_2$ by virtue of

$$\varepsilon_2 \varepsilon_3 = \frac{1}{2}\left(-1 + i\sqrt{3}\right) \cdot \frac{1}{2}\left(-1 - i\sqrt{3}\right) = \frac{1}{4}(1+3) = 1\,.$$

Presuming that the radicand of the square root is nonnegative,

$$\Delta = \left(\frac{q}{2}\right)^2 + \left(\frac{p}{3}\right)^3 \geq 0\,,$$

the solution y_1 is real,

$$y_1 = u_1 + v_1 = \sqrt[3]{-\frac{q}{2} + \sqrt{\Delta}} + \sqrt[3]{-\frac{q}{2} - \sqrt{\Delta}}\,, \tag{SM-4.10}$$

while y_2 and y_3 are conjugate complex, as can be seen below:

$$\begin{aligned} y_2 &= u_2 + v_3 = u_1\varepsilon_2 + v_1\varepsilon_3 = -\frac{1}{2}(u_1 + v_1) + \left[\frac{1}{2}(u_1 - v_1)\right] \cdot i\sqrt{3}\,, \\ y_3 &= u_3 + v_2 = u_1\varepsilon_3 + v_1\varepsilon_2 = -\frac{1}{2}(u_1 + v_1) - \left[\frac{1}{2}(u_1 - v_1)\right] \cdot i\sqrt{3}\,. \end{aligned} \tag{SM-4.11}$$

The solution given by Equation (SM-4.10) is known as Cardano's formula.

For the case that the radicand of the square root is negative ("casus irreducibilis"),

$$\Delta = \left(\frac{q}{2}\right)^2 + \left(\frac{p}{3}\right)^3 < 0\,,$$

there exist three real trigonometric solutions for y in Equation(SM-4.3). Introducing the auxiliary variables

$$r_0 = \sqrt{-\left(\frac{p}{3}\right)^3}\,, \quad \cos\varphi = -\frac{q}{2\sqrt{-\left(\frac{p}{3}\right)^3}}\,,$$

these three real solutions read:

$$\begin{aligned} y_1 &= 2\sqrt[3]{r_0}\cos\left(\frac{\varphi}{3}\right)\,, \\ y_2 &= 2\sqrt[3]{r_0}\cos\left(\frac{\varphi}{3}+\frac{2\pi}{3}\right)\,, \\ y_3 &= 2\sqrt[3]{r_0}\cos\left(\frac{\varphi}{3}+\frac{4\pi}{3}\right)\,. \end{aligned} \tag{SM-4.12}$$

The back transformation from the solutions of the reduced form, y_i, to the solutions of the normal form, x_i, is carried out using Equation (SM-4.3).

SM-5 Table Values of the Computer-Program Verification

Table SM-5.1. Deviations of the numerically determined values of mass density $\widehat{\varrho}$, thermal expansion coefficient α_p, isothermal compressibility κ_T, isobaric heat capacity c_p, speed of sound w, equilibrium mole fraction of low-density water, x_e, and the ordering field L using the root finder of Press *et al.* [31] (Section 9.1 therein) from the IAPWS G12-15 [1] reference values (subscript $\star$, Table SM-3.5.1). Integer n denotes the number of equally spaced segments of the root interval of x_e. Relative deviations are given in parts per billion (ppb). Bold-styled values denote extrema.

$\frac{T}{\text{K}}$	$\frac{p}{\text{MPa}}$	$\frac{\widehat{\varrho}-\widehat{\varrho}_\star}{\widehat{\varrho}_\star}$	$\frac{\alpha_p-\alpha_{p,\star}}{10^{-4}\,\text{K}^{-1}}$	$\frac{\kappa_T-\kappa_{T,\star}}{\kappa_{T,\star}}$	$\frac{\widehat{c}_p-\widehat{c}_{p,\star}}{\widehat{c}_{p,\star}}$	$\frac{w-w_\star}{w_\star}$	$\frac{x_e-x_{e\star}}{x_{e,\star}}$	$\frac{L-L_\star}{L_\star}$
				n = 10^9				
273.15	0.101325	1.40	$-$ **0.49·10^{-6}**	91.40	−7.36	−25.29	0.65	−4.24
235.15	0.101325	**−3.23**	−0.28·10^{-6}	34.53	8.01	**−29.12**	0.09	**−45.23**
250	200	−1.88	0.11·10^{-6}	−45.28	**−10.95**	−21.75	1.14	−3.61
200	400	−2.64	−0.42·10^{-6}	**−135.49**	−7.67	−0.18	**3.32**	−29.13
250	400	2.32	0.31·10^{-6}	109.62	9.59	−20.41	3.30	−11.59
				n = 10^8				
273.15	0.101325	2.21	−0.31·10^{-6}	82.11	−10.15	−21.20	**−12.53**	
235.15	0.101325	**−2.87**	0.36·10^{-7}	29.06	3.03	**−29.00**	−3.28	
250	200	−1.70	0.14·10^{-6}	−49.39	**−10.98**	−19.60	−5.46	
200	400	−2.64	**−0.42·10^{-6}**	**−135.41**	−7.67	−0.23	3.62	
250	400	2.32	0.31·10^{-6}	109.65	9.58	−20.42	3.40	
				n = 10^7				
273.15	0.101325	3.63	0.93·10^{-8}	65.78	−15.04	−14.01	−35.71	
235.15	0.101325	**−9.44**	**−0.56·10^{-5}**	127.69	**92.79**	**31.16**	**57.56**	
250	200	−2.21	0.70·10^{-7}	−37.68	−10.90	−25.73	13.32	
200	400	−2.66	−0.42·10^{-6}	**−134.63**	−7.67	−0.65	6.35	
250	400	2.33	0.31·10^{-6}	109.08	9.59	−20.12	0.93	
				n = 10^6				
273.15	0.101325	17.92	0.32·10^{-5}	−98.61	−64.31	**58.32**	−269.00	
235.15	0.101325	**57.40**	**0.52·10^{-4}**	**−875.69**	**−820.26**	−9.13	**−561.33**	
250	200	−0.37	0.31·10^{-6}	−79.81	−11.19	−3.70	−54.21	
200	400	−2.23	−0.41·10^{-6}	−150.79	−7.54	8.11	−50.74	
250	400	1.95	0.29·10^{-6}	122.99	9.46	−27.61	61.19	

Continuation of Table SM-5.1.

$\frac{T}{\mathrm{K}}$	$\frac{p}{\mathrm{MPa}}$	$\frac{\widehat{\varrho}-\widehat{\varrho}_\star}{\widehat{\varrho}_\star}$	$\frac{\alpha_p-\alpha_{p,\star}}{10^{-4}\,\mathrm{K}^{-1}}$	$\frac{\kappa_T-\kappa_{T,\star}}{\kappa_{T,\star}}$	$\frac{\widehat{c}_p-\widehat{c}_{p,\star}}{\widehat{c}_{p,\star}}$	$\frac{w-w_\star}{w_\star}$	$\frac{x_e-x_{e\star}}{x_{e,\star}}$	$\frac{L-L_\star}{L_\star}$
				n = 10^5				
273.15	0.101325	−124.98	$-0.29\cdot10^{-4}$	1545.41	428.40	**−665.03**	2064.05	
235.15	0.101325	**−801.76**	$\mathbf{-0.68\cdot10^{-3}}$	**12022.59**	**10916.90**	−292.31	**7394.27**	
250	200	17.99	$0.27\cdot10^{-5}$	−501.08	−14.16	216.56	−729.56	
200	400	−3.66	$-0.44\cdot10^{-6}$	−96.93	−7.98	−21.07	139.56	
250	400	2.37	$0.31\cdot10^{-6}$	107.56	9.60	−19.30	−5.67	
				n = 10^4				
273.15	0.101325	4162.02	$0.93\cdot10^{-3}$	−47775.34	−14352.32	**21036.79**	−67927.36	
235.15	0.101325	**7790.13**	$\mathbf{0.67\cdot10^{-2}}$	**−116951.57**	**−106440.33**	2543.51	**−72161.87**	
250	200	1670.25	$0.22\cdot10^{-3}$	−38414.77	−281.40	20040.61	−61509.86	
200	400	39.29	$0.46\cdot10^{-6}$	−1712.79	5.15	854.42	−5569.52	
250	400	−10.22	$-0.40\cdot10^{-6}$	570.43	5.18	−268.67	1999.93	
				n = 10^3				
273.15	0.101325	47036.98	$0.11\cdot10^{-1}$	−540984.39	−162098.23	**238206.89**	−767841.48	
235.15	0.101325	**93738.95**	$\mathbf{0.80\cdot10^{-1}}$	**−1405616.97**	**−1278231.17**	31390.30	**−867723.32**	
250	200	−165.60	$-0.21\cdot10^{-4}$	3711.58	15.53	−1986.04	6023.81	
200	400	373.38	$0.74\cdot10^{-5}$	−14280.55	107.24	7663.89	−49973.49	
250	400	−52.19	$-0.28\cdot10^{-5}$	2113.33	−9.56	−1099.92	8685.26	
				n = 10^2				
273.15	0.101325	190018.18	$0.42\cdot10^{-1}$	−2185035.19	−653781.33	964106.57	−3100888.54	
235.15	0.101325	**380627.98**	**0.32**	**−5687105.11**	**−5160921.64**	133965.74	−3519594.82	
250	200	165107.11	$0.22\cdot10^{-1}$	−3785627.41	−26483.21	**1985851.69**	**−6072006.33**	
200	400	850.66	$0.17\cdot10^{-4}$	−32234.42	253.09	17391.91	−113407.72	
250	400	−3829.77	$-0.22\cdot10^{-3}$	140976.82	−1336.42	−75904.32	610364.54	
				n = 10^1				
273.15	0.101325	**−12287514.62**	**−2.95**	**145898351.27**	**48896156.92**	**−53603509.82**	**206873347.01**	
235.15	0.101325	1341300.94	1.13	−19805072.01	−17848920.22	547357.55	−12359166.48	
250	200	1823017.78	0.24	−41455907.74	−266681.45	22491481.74	−66852307.68	
200	400	−32557.06	$-0.68\cdot10^{-3}$	1224714.96	−9955.36	−662964.78	4326988.81	
250	400	−8027.03	$-0.46\cdot10^{-3}$	295274.11	−2810.64	−159002.41	1278897.07	

SM-6 Table Values of the Deviations of the Analytical from the IAPWS G12-15 [1] Reference Formulation

Table SM-6.1. Deviation of the analytically determined mass density $\widehat{\varrho}$, thermal expansion coefficient α_p, isothermal compressibility κ_T, isobaric heat capacity c_p, speed of sound w, equilibrium mole fraction of low-density water, x_e, and the ordering field L according to Section SM-3.1 from the IAPWS G12-15 [1] reference values (subscript $\star$, Table SM-3.5.1). Relative deviations are given in parts per billion (ppb). Bold-styled values denote extrema.

$\frac{T}{\mathrm{K}}$	$\frac{p}{\mathrm{MPa}}$	$\frac{\widehat{\varrho}-\widehat{\varrho}_\star}{\widehat{\varrho}_\star}$	$\frac{\alpha_p-\alpha_{p,\star}}{10^{-4}\,\mathrm{K}^{-1}}$	$\frac{\kappa_T-\kappa_{T,\star}}{\kappa_{T,\star}}$	$\frac{\widehat{c}_p-\widehat{c}_{p,\star}}{\widehat{c}_{p,\star}}$	$\frac{w-w_\star}{w_\star}$	$\frac{x_e-x_{e\star}}{x_{e,\star}}$	$\frac{L-L_\star}{L_\star}$
$n = 10^9$								
273.15	0.101325	2.20	$-0.31{\cdot}10^{-6}$	82.19	-10.13	-21.23	-12.43	-4.24
235.15	0.101325	$\mathbf{0.172{\cdot}10^{6}}$	**0.15**	$\mathbf{-2.58{\cdot}10^{6}}$	$\mathbf{-2.34{\cdot}10^{6}}$	$\mathbf{0.059{\cdot}10^{6}}$	$\mathbf{-1.596{\cdot}10^{6}}$	$\mathbf{-45.23}$
250	200	-1.88	$0.11{\cdot}10^{-6}$	-45.26	-10.95	-21.76	1.16	3.61
200	400	-2.64	$-0.42{\cdot}10^{-6}$	-135.50	-7.67	-0.17	3.28	29.13
250	400	2.32	$0.31{\cdot}10^{-6}$	109.63	9.59	-20.41	3.31	-11.59

SM-7 Table Values of the Deviation of the Analytically from the Numerically Determined Thermodynamic Properties of Subcooled Water

SM-7.1 Mass Density

Table SM-7.1.1. Relative deviation, $(\widehat{\varrho}-\widehat{\varrho}_{\mathrm{num}})/\widehat{\varrho}_{\mathrm{num}}$ in parts per billion (ppb), of the mass density $\widehat{\varrho}$ using the analytically determined low-density water fraction from the mass density $\widehat{\varrho}_{\mathrm{num}}$ using the numerically determined low-density water fraction at p=0.101325 MPa.

$\frac{T}{\mathrm{K}}$	$\frac{\widehat{\varrho}-\widehat{\varrho}_{\mathrm{num}}}{\widehat{\varrho}_{\mathrm{num}}}$
235.15	**172468.89**
240.15	19920.96
245.15	3096.26
250.15	573.01
255.15	119.43
260.15	26.98
265.15	6.44
270.15	1.78
275.15	0.38
280.15	0.13
285.15	0.03
290.15	−0.03
295.15	−0.06
300.15	0.13

Table SM-7.1.2. As Table SM-7.1.1, but for $0.1 \leq p/\mathrm{MPa} \leq 400$ and $253.15 \leq T/\mathrm{K} \leq 303.15$.

$\frac{p}{\mathrm{MPa}}$	T/K											
	253.15	255.65	258.15	260.65	263.15	265.65	268.15	270.65	273.15	293.15	298.15	303.15
0.1	**220.99**	102.65	48.57	23.44	11.49	5.61	2.95	1.54	0.79	−0.10	−0.13	0.05
5.0	152.68	71.77	34.23	16.71	8.19	4.22	2.09	1.14	0.49	0.12	0.03	0.00
10.0	104.40	49.70	23.89	11.95	5.94	2.97	1.52	0.76	0.52	−0.15	0.10	0.12
15.0	71.10	34.15	16.66	8.43	4.05	2.00	1.18	0.57	0.29	−0.02	−0.15	−0.00
20.0	48.26	23.40	11.72	5.86	3.10	1.59	0.66	0.38	0.29	−0.11	0.00	−0.01
25.0	32.52	16.07	8.06	4.13	2.07	1.03	0.44	0.24	0.26	−0.02	−0.09	−0.01
30.0	22.14	10.94	5.66	2.70	1.55	0.68	0.28	0.19	0.07	0.08	−0.01	−0.01
35.0	14.96	7.38	3.78	1.91	1.15	0.50	0.35	0.04	0.01	0.01	0.01	−0.00
40.0	9.99	5.17	2.63	1.45	0.79	0.27	0.24	0.14	0.14	−0.12	−0.01	−0.01
45.0	6.75	3.33	1.70	0.86	0.54	0.42	0.26	0.00	−0.07	−0.01	−0.01	−0.01
50.0	4.56	2.30	1.13	0.51	0.21	0.19	0.02	0.09	0.10	0.01	0.01	0.00
55.0	2.98	1.55	0.84	0.30	0.24	0.19	0.20	0.15	−0.13	−0.01	0.00	0.01
60.0	2.04	1.05	0.52	0.24	0.08	0.12	−0.07	0.05	−0.08	0.00	−0.01	−0.01
65.0	1.48	0.87	0.53	0.36	0.22	0.10	0.12	0.12	−0.09	−0.01	0.01	−0.01
70.0	0.76	0.36	0.34	0.25	0.18	0.15	−0.01	0.12	−0.08	−0.01	−0.01	−0.01
75.0	0.66	0.24	0.11	0.03	−0.02	−0.04	0.14	0.14	0.01	−0.01	−0.01	−0.01
80.0	0.47	0.07	0.13	0.02	0.19	0.02	−0.05	0.14	0.14	−0.02	−0.01	0.01
85.0	0.12	0.23	0.19	0.21	−0.13	0.10	0.01	−0.11	−0.16	0.01	−0.02	−0.00
90.0	0.18	0.02	0.02	−0.08	−0.05	0.09	−0.07	0.17	−0.12	−0.01	−0.02	−0.00
95.0	−0.04	0.03	0.09	−0.13	0.14	−0.11	0.05	−0.03	−0.00	−0.01	−0.01	0.00
100.0	0.13	0.00	0.15	0.04	0.03	0.15	−0.05	0.00	−0.01	0.01	−0.01	0.01
105.0	−0.00	−0.06	0.04	0.03	0.17	0.12	−0.00	0.00	0.02	0.01	−0.00	−0.00
110.0	0.05	−0.04	−0.10	−0.12	0.10	−0.01	0.00	−0.01	−0.01	−0.01	−0.01	0.01

Continuation of Table SM-7.1.2.

$\frac{p}{\text{MPa}}$	T/K											
	253.15	255.65	258.15	260.65	263.15	265.65	268.15	270.65	273.15	293.15	298.15	303.15
115.0	−0.08	0.03	0.14	−0.05	0.02	0.01	0.01	−0.00	−0.01	−0.01	−0.00	−0.01
120.0	0.15	0.00	0.16	−0.01	−0.01	0.00	−0.01	−0.00	−0.01	0.00	0.02	0.00
125.0	−0.16	−0.07	0.01	0.00	0.01	0.00	−0.02	−0.01	0.01	−0.00	−0.01	−0.01
130.0	−0.07	0.00	−0.01	0.00	−0.01	−0.01	−0.01	0.01	−0.01	0.00	−0.01	0.01
135.0	0.02	0.00	−0.01	0.01	−0.01	−0.01	0.00	−0.00	−0.00	−0.00	0.02	0.00
140.0	0.00	0.00	−0.01	0.00	0.00	0.02	−0.01	0.02	0.01	0.01	0.01	0.01
145.0	−0.00	−0.01	0.01	−0.01	0.00	0.00	−0.00	0.00	−0.01	0.01	0.01	−0.00
150.0	−0.01	−0.00	−0.01	−0.02	0.01	−0.00	−0.01	−0.00	−0.00	−0.01	−0.01	−0.01
155.0	0.01	0.01	−0.01	0.02	0.01	0.01	−0.01	0.02	−0.01	0.00	0.01	0.01
160.0	0.00	−0.00	−0.01	−0.01	−0.00	0.00	0.00	−0.01	−0.00	−0.01	0.01	0.01
165.0	0.01	0.01	0.00	0.02	−0.01	−0.01	0.00	0.01	0.01	−0.00	−0.01	0.00
170.0	−0.01	−0.01	−0.00	0.01	−0.01	−0.01	0.02	0.00	0.00	0.01	−0.01	−0.00
175.0	−0.01	−0.02	−0.01	−0.01	0.01	0.01	0.01	−0.01	0.00	−0.00	0.02	0.01
180.0	0.01	−0.02	−0.01	0.01	0.01	0.01	0.01	−0.01	−0.01	0.02	−0.01	0.01
185.0	−0.02	0.01	0.00	−0.00	0.00	−0.02	0.01	−0.01	−0.01	0.01	−0.01	0.01
190.0	0.01	−0.00	0.00	0.01	−0.00	0.00	0.01	−0.01	−0.00	0.01	0.01	−0.00
195.0	0.01	0.02	0.01	−0.01	−0.02	−0.02	0.01	0.01	−0.01	0.01	−0.00	0.01
200.0	−0.01	0.01	−0.01	−0.01	0.01	−0.02	0.01	0.02	0.01	−0.01	−0.01	−0.01
205.0	0.02	−0.01	0.01	0.00	0.00	0.02	−0.00	0.01	0.02	0.01	−0.00	0.01
210.0	−0.01	0.01	−0.00	−0.01	0.01	0.01	−0.00	−0.01	−0.00	0.01	0.01	−0.00
215.0	−0.00	−0.01	−0.01	0.01	−0.01	0.02	0.02	0.01	0.00	0.02	0.02	−0.01
220.0	−0.00	0.01	0.01	−0.00	0.02	0.02	0.01	0.01	−0.01	−0.00	0.02	−0.02
225.0	−0.01	0.00	0.01	0.00	0.01	−0.02	0.01	−0.01	−0.01	0.00	−0.02	0.01
230.0	−0.01	−0.01	−0.01	−0.01	−0.01	−0.02	0.02	0.00	0.00	−0.01	−0.01	−0.00
235.0	0.02	−0.01	0.01	−0.00	−0.01	−0.02	−0.00	−0.01	0.01	0.02	0.00	0.01
240.0	−0.01	−0.00	0.01	0.01	−0.00	−0.02	0.02	−0.00	−0.01	0.02	0.01	−0.01
245.0	−0.01	0.00	−0.00	−0.01	−0.00	−0.00	−0.02	0.00	−0.01	0.02	−0.00	−0.01
250.0	0.02	−0.00	−0.00	0.02	−0.01	0.02	−0.01	0.01	0.01	−0.01	−0.02	0.01

Continuation of Table SM-7.1.2.

$\frac{p}{\text{MPa}}$	T/K											
	253.15	255.65	258.15	260.65	263.15	265.65	268.15	270.65	273.15	293.15	298.15	303.15
255.0	−0.02	0.02	−0.02	−0.00	−0.01	0.01	0.02	0.02	−0.02	0.01	0.00	0.01
260.0	−0.02	−0.00	0.02	−0.00	−0.02	0.02	−0.00	−0.00	−0.02	0.00	0.00	−0.00
265.0	0.02	−0.01	0.00	0.01	−0.01	−0.00	−0.01	0.02	0.01	0.00	0.02	0.00
270.0	0.02	−0.02	−0.01	0.00	0.01	−0.02	0.01	−0.01	−0.01	0.02	−0.00	−0.00
275.0	−0.01	−0.02	−0.02	0.02	−0.00	−0.00	0.02	−0.01	0.01	−0.01	−0.00	0.00
280.0	0.00	−0.00	0.02	−0.01	−0.01	0.02	−0.00	−0.00	−0.01	0.00	0.00	−0.00
285.0	−0.01	−0.01	−0.00	−0.00	−0.01	−0.00	0.02	−0.00	0.01	0.00	0.00	−0.00
290.0	0.01	0.01	−0.00	0.01	−0.01	0.02	0.00	−0.00	−0.01	−0.00	−0.00	−0.00
295.0	−0.01	−0.01	0.01	−0.00	0.01	−0.02	−0.01	0.02	0.00	0.00	−0.00	0.00
300.0	−0.02	0.02	0.01	0.01	−0.01	0.00	−0.02	−0.01	−0.02	0.00	−0.00	−0.00
305.0	−0.02	−0.02	0.01	−0.01	0.01	−0.01	−0.00	0.00	0.00	0.00	0.00	−0.00
310.0	−0.00	−0.01	−0.01	0.02	−0.00	−0.02	−0.02	−0.00	0.00	0.00	0.00	−0.00
315.0	0.02	0.01	−0.02	0.01	−0.01	0.00	0.00	−0.00	−0.00	0.00	−0.00	−0.00
320.0	−0.01	0.02	−0.01	−0.00	−0.00	0.00	0.00	0.00	0.00	0.00	0.00	−0.00
325.0	0.00	0.00	0.00	−0.00	−0.00	0.00	−0.00	−0.00	0.00	0.00	0.00	−0.00
330.0	0.00	−0.00	−0.00	0.00	−0.00	−0.00	0.00	−0.00	0.00	−0.00	−0.00	−0.00
335.0	0.00	−0.00	0.00	0.00	−0.00	0.00	−0.00	0.00	−0.00	−0.00	−0.00	−0.00
340.0	0.00	0.00	−0.00	−0.00	0.00	−0.00	−0.00	−0.00	−0.00	−0.00	−0.00	−0.00
345.0	−0.00	0.00	0.00	0.00	0.00	0.00	0.00	0.00	−0.00	0.00	0.00	0.00
350.0	−0.00	0.00	−0.00	0.00	0.00	−0.00	0.00	−0.00	−0.00	−0.00	0.00	0.00
355.0	−0.00	0.00	0.00	0.00	0.00	−0.00	0.00	−0.00	−0.00	0.00	0.00	−0.00
360.0	−0.00	−0.00	0.00	0.00	0.00	0.00	−0.00	−0.00	0.00	−0.00	0.00	0.00
365.0	0.00	0.00	0.00	0.00	−0.00	−0.00	0.00	0.00	0.00	0.00	−0.00	−0.00
370.0	−0.00	−0.00	−0.00	0.00	−0.00	0.00	−0.00	−0.00	0.00	0.00	−0.00	0.00
375.0	−0.00	−0.00	−0.00	0.00	0.00	0.00	−0.00	−0.00	0.00	0.00	0.00	0.00
380.0	0.00	−0.00	0.00	−0.00	−0.00	0.00	0.00	0.00	0.00	−0.00	0.00	0.00

Continuation of Table SM-7.1.2.

$\frac{p}{\text{MPa}}$	T/K											
	253.15	255.65	258.15	260.65	263.15	265.65	268.15	270.65	273.15	293.15	298.15	303.15
385.0	0.00	−0.00	0.00	0.00	0.00	0.00	−0.00	0.00	0.00	−0.00	−0.00	−0.00
390.0	0.00	−0.00	0.00	−0.00	0.00	−0.00	−0.00	−0.00	0.00	−0.00	−0.00	−0.00
395.0	0.00	0.00	−0.00	0.00	0.00	−0.00	0.00	−0.00	0.00	−0.00	0.00	−0.00
400.0	−0.00	0.00	−0.00	−0.00	−0.00	0.00	0.00	0.00	−0.00	−0.00	−0.00	0.00

Table SM-7.1.3. As Table SM-7.1.1, but for $0.1 \leq p/\mathrm{MPa} \leq 1000$ and $235.15 \leq T/\mathrm{K} \leq 303.15$.

p/MPa	T/K			
	273.15	293.15	298.15	303.15
0.1	**0.79**	−0.10	−0.13	0.05
50.0	0.10	0.01	0.01	0.00
100.0	−0.01	0.01	−0.01	0.01
150.0	−0.00	−0.01	−0.01	−0.01
200.0	0.01	−0.01	−0.01	−0.01
250.0	0.01	−0.01	−0.02	0.01
300.0	−0.02	0.00	−0.00	−0.00
350.0	−0.00	−0.00	0.00	0.00
400.0	−0.00	−0.00	−0.00	0.00
450.0	0.00	−0.00	−0.00	0.00
500.0	−0.00	0.00	−0.00	−0.00
550.0	−0.00	0.00	−0.00	−0.00
600.0	−0.00	0.00	−0.00	0.00
650.0	0.00	0.00	0.00	0.00
700.0	−0.00	0.00	0.00	−0.00
750.0	−0.00	0.00	−0.00	−0.00
800.0	−0.00	−0.00	0.00	−0.00
850.0	0.00	−0.00	−0.00	0.00
900.0	0.00	−0.00	0.00	0.00
950.0	−0.00	0.00	−0.00	−0.00
1000.0	−0.00	0.00	−0.00	0.00

Table SM-7.1.4. As Table SM-7.1.1, but for $200 \leq p/\text{hPa} \leq 1000$ and $235.15 \leq T/K \leq 300.15$.

T/K	p/hPa								
	1000	900	800	700	600	500	400	300	200
235.15	172494.11	172684.18	172874.63	173065.06	173255.86	173446.84	173638.00	173829.53	**174021.05**
240.15	19923.63	19942.33	19961.02	19979.90	19998.77	20017.41	20036.25	20055.29	20074.10
245.15	3096.52	3099.25	3101.81	3104.41	3107.06	3109.77	3112.29	3115.09	3117.71
250.15	572.97	573.37	573.85	574.42	574.82	575.31	575.64	576.06	576.56
255.15	119.34	119.46	119.59	119.47	119.60	119.73	119.87	120.00	120.14
260.15	26.97	27.21	27.03	27.22	27.27	27.17	27.18	27.30	27.27
265.15	6.54	6.59	6.56	6.47	6.57	6.59	6.54	6.69	6.49
270.15	1.72	1.58	1.71	1.82	1.63	1.71	1.78	1.83	1.58
275.15	0.31	0.57	0.58	0.33	0.40	0.50	0.34	0.50	0.41
280.15	0.09	0.05	0.08	0.18	0.05	−0.01	0.01	0.09	0.25
285.15	−0.10	−0.01	0.18	0.17	−0.03	0.17	0.17	−0.02	−0.11
290.15	0.13	−0.12	0.08	0.10	−0.06	−0.09	0.02	−0.05	0.01
295.15	−0.13	−0.02	−0.08	0.03	−0.03	0.07	0.02	0.12	0.06
300.15	−0.14	−0.03	−0.06	0.09	0.09	−0.04	0.00	−0.10	−0.02

SM-7.2 Thermal Expansivity

Table SM-7.2.1. Deviation, $(\alpha_p - \alpha_{p,\mathrm{num}}) / (10^{-4}\,\mathrm{K}^{-1})$, of the thermal expansivity α_p using the analytically determined low-density water fraction from the thermal expansivity $\alpha_{p,\mathrm{num}}$ using the numerically determined low-density water fraction for $0.1 \leq p/\mathrm{MPa} \leq 600$ and $245.5 \leq T/\mathrm{K} \leq 288.0$.

$\frac{p}{\mathrm{MPa}}$	T/K					
	245.5	253.2	262.8	273.8	281.3	288.0
0.1	**0.11E-02**	0.68E-04	0.32E-05	0.16E-06	0.16E-07	0.65E-08
10.0	0.44E-03	0.31E-04	0.16E-05	0.81E-07	0.91E-08	0.31E-08
20.0	0.17E-03	0.14E-04	0.79E-06	0.49E-07	0.11E-07	0.14E-07
30.0	0.67E-04	0.59E-05	0.39E-06	0.24E-07	0.24E-07	-0.12E-07
40.0	0.26E-04	0.25E-05	0.18E-06	-0.14E-07	-0.52E-08	-0.84E-09
50.0	0.99E-05	0.10E-05	0.11E-06	-0.15E-07	-0.23E-07	0.98E-09
60.0	0.38E-05	0.47E-06	0.17E-07	0.16E-07	-0.29E-07	0.24E-08
70.0	0.15E-05	0.23E-06	0.20E-08	-0.35E-08	0.30E-07	-0.25E-08
80.0	0.54E-06	0.71E-07	0.10E-07	0.49E-08	-0.11E-08	0.14E-08
90.0	0.21E-06	0.70E-08	0.35E-07	0.27E-07	0.18E-08	-0.48E-09
100.0	0.12E-06	0.43E-07	-0.13E-08	-0.18E-09	-0.82E-09	0.24E-09
110.0	0.21E-07	-0.24E-07	-0.18E-08	0.12E-08	-0.19E-08	-0.24E-08
120.0	0.26E-07	-0.11E-07	0.25E-08	0.22E-08	0.68E-09	0.14E-09
130.0	-0.21E-09	-0.18E-07	0.77E-10	-0.96E-09	0.21E-09	-0.17E-08
140.0	0.73E-08	0.92E-09	-0.77E-10	-0.22E-08	0.71E-09	-0.20E-08
150.0	0.18E-08	0.19E-08	-0.80E-09	0.47E-10	0.25E-08	0.80E-09
160.0	-0.42E-09	-0.15E-09	-0.26E-08	-0.20E-08	-0.19E-08	0.25E-08
170.0	-0.18E-08	0.18E-08	-0.21E-08	0.23E-08	-0.24E-08	0.22E-08
180.0	0.22E-08	-0.32E-09	-0.20E-08	-0.22E-08	-0.26E-08	-0.19E-09
190.0	-0.13E-08	0.18E-08	-0.65E-09	-0.22E-09	-0.86E-09	0.11E-08
200.0	-0.38E-09	-0.13E-08	0.79E-09	-0.21E-08	0.76E-09	-0.14E-08
210.0	0.21E-08	0.33E-09	-0.16E-08	0.22E-09	0.18E-08	0.10E-09
220.0	-0.21E-08	-0.21E-08	0.16E-08	0.99E-09	0.34E-09	-0.17E-08
230.0	0.63E-09	-0.20E-08	-0.20E-08	-0.18E-08	0.13E-08	0.16E-08
240.0	-0.49E-09	0.22E-08	0.19E-08	0.61E-09	0.25E-08	0.16E-08
250.0	-0.45E-09	0.32E-09	0.19E-08	-0.57E-10	-0.77E-09	0.20E-08
260.0	-0.15E-09	0.29E-09	-0.10E-08	0.99E-10	0.68E-09	-0.68E-09
270.0	0.18E-08	0.50E-09	-0.18E-08	-0.23E-08	-0.19E-08	-0.18E-08
280.0	-0.12E-08	0.11E-08	-0.19E-08	-0.10E-08	-0.14E-08	-0.12E-08
290.0	-0.11E-08	0.15E-08	0.27E-09	-0.12E-08	-0.23E-08	-0.47E-10
300.0	0.17E-08	-0.64E-09	-0.67E-09	-0.35E-09	0.54E-10	0.43E-10
310.0	0.48E-09	-0.12E-08	-0.66E-09	-0.16E-10	0.62E-11	0.45E-10
320.0	-0.70E-09	-0.12E-08	0.22E-10	-0.24E-10	-0.50E-10	-0.29E-10
330.0	-0.38E-09	-0.51E-10	-0.38E-10	-0.52E-10	-0.43E-11	0.35E-10
340.0	-0.65E-11	-0.43E-10	0.47E-10	0.49E-10	-0.42E-10	0.38E-10
350.0	0.22E-11	0.45E-11	-0.30E-10	-0.88E-11	-0.30E-10	0.39E-11
360.0	0.69E-11	-0.31E-10	0.12E-10	-0.24E-10	-0.52E-11	0.24E-10
370.0	0.13E-10	0.13E-10	0.31E-10	0.45E-11	-0.12E-10	0.39E-11
380.0	-0.26E-10	-0.23E-10	-0.23E-10	0.17E-10	-0.28E-10	-0.31E-10
390.0	0.13E-11	-0.18E-10	0.23E-10	-0.25E-10	-0.64E-11	-0.14E-11
400.0	0.47E-11	0.19E-10	0.20E-10	0.36E-11	0.99E-11	0.32E-11
410.0	-0.68E-11	-0.12E-10	-0.18E-10	0.63E-11	0.14E-10	0.33E-11
420.0	0.15E-10	-0.16E-10	-0.77E-11	0.15E-10	-0.14E-10	-0.12E-10
430.0	-0.53E-11	-0.11E-10	-0.43E-11	0.12E-10	0.31E-11	0.10E-11
440.0	0.11E-10	0.10E-10	0.14E-10	-0.57E-11	-0.17E-10	0.88E-11
450.0	0.55E-11	0.79E-11	0.68E-11	0.68E-11	0.19E-11	0.57E-11
460.0	-0.22E-11	-0.90E-11	-0.54E-11	0.12E-10	0.10E-10	0.14E-10
470.0	0.44E-13	-0.38E-11	-0.43E-11	0.13E-10	0.81E-11	-0.10E-10
480.0	-0.32E-11	-0.86E-11	-0.12E-11	0.28E-11	-0.95E-11	0.68E-11
490.0	-0.62E-11	-0.72E-11	-0.27E-11	0.81E-11	0.10E-10	-0.11E-10

Continuation of Table SM-7.2.1.

$\frac{p}{\text{MPa}}$	T/K					
	245.5	253.2	262.8	273.8	281.3	288.0
500.0	0.51E-11	-0.18E-11	-0.60E-11	-0.20E-11	-0.66E-11	0.68E-11
510.0	-0.12E-11	-0.48E-11	0.21E-11	-0.37E-11	0.41E-11	0.71E-11
520.0	0.30E-11	-0.41E-11	-0.54E-12	-0.42E-11	0.59E-11	-0.33E-11
530.0	0.29E-11	-0.34E-11	0.48E-13	-0.81E-12	0.25E-11	0.21E-11
540.0	-0.14E-11	0.34E-11	0.65E-14	0.40E-11	0.29E-11	0.34E-11
550.0	0.89E-12	0.98E-12	-0.38E-11	-0.46E-11	-0.37E-11	0.28E-11
560.0	0.14E-11	-0.24E-11	-0.32E-11	-0.98E-12	-0.44E-11	0.47E-11
570.0	-0.10E-11	-0.75E-13	-0.66E-12	-0.20E-11	-0.30E-11	0.35E-11
580.0	-0.11E-14	-0.47E-12	0.27E-12	-0.23E-11	-0.11E-11	-0.26E-11
590.0	-0.96E-12	0.21E-12	-0.16E-11	0.97E-12	0.15E-11	-0.58E-12
600.0	-0.88E-12	-0.12E-11	-0.93E-12	-0.69E-12	-0.32E-12	0.17E-11

155 ## SM-7.3 Compressibility

Table SM-7.3.1. Relative deviation, $(\kappa_T - \kappa_{T,\mathrm{num}})/\kappa_{T,\mathrm{num}}$ in parts per billion (ppb), of the isothermal compressibility κ_T using the analytically determined low-density water fraction from the thermal expansivity $\kappa_{T,\mathrm{num}}$ using the numerically determined low-density water fraction for $0.1 \leq p/\mathrm{MPa} \leq 190$ and $235.15 \leq T/\mathrm{K} \leq 300.15$.

T/K	p/MPa					
	0.101325	10.0	50.0	100.0	150.0	190.0
235.15	**−2583606.23**	−868662.57	−12123.11	−86.13	−3.93	−0.25
240.15	−257131.42	−104712.54	−2534.75	−23.65	1.62	−0.25
245.15	−37519.10	−16968.92	−589.71	−6.69	0.17	−0.01
250.15	−6743.93	−3268.78	−149.13	−5.37	−0.30	−0.37
255.15	−1386.73	−705.53	−40.33	−2.79	−0.31	−0.03
260.15	−311.39	−165.93	−9.89	−2.20	0.15	0.36
265.15	−74.12	−40.47	−4.30	2.64	0.30	0.30
270.15	−20.46	−9.19	0.30	0.13	0.01	0.20
275.15	−4.39	−2.04	−0.40	0.25	−0.30	0.16
280.15	−1.45	−1.92	−0.75	−0.24	0.09	0.13
285.15	−0.36	1.46	2.08	0.07	0.00	−0.17
290.15	0.38	−1.44	1.76	0.13	0.25	−0.19
295.15	0.70	1.35	0.17	−0.01	−0.22	0.31
300.15	−1.50	0.38	−0.12	0.20	−0.12	−0.22

SM-7.4 Isobaric Heat Capacity

Table SM-7.4.1. Relative deviation, $\left(\hat{c}_p - \hat{c}_{p,\mathrm{num}}\right) / \hat{c}_{p,\mathrm{num}}$ in parts per billion (ppb), of the isobaric heat capacity $\hat{c}_p$ using the analytically determined low-density water fraction from the isobaric heat capacity $\hat{c}_{p,\mathrm{num}}$ using the numerically determined low-density water fraction for $0.1 \leq p/\mathrm{MPa} \leq 190$ and $235.15 \leq T/\mathrm{K} \leq 300.15$.

T/K	p/MPa							
	0.101325	19.6	29.4	39.2	49.0	68.6	98.1	150.0	190.0
235.15	−**2348083.25**	−173405.05	−48054.63	−13614.26	−3938.60	−348.45	−10.17	−0.11	−0.00
240.15	−180561.64	−19872.54	−6502.75	−2131.01	−700.89	−76.96	−2.72	0.05	−0.00
245.15	−21310.20	−2984.89	−1091.15	−395.69	−142.83	−18.92	−1.09	0.01	−0.00
250.15	−3219.47	−533.75	−211.66	−83.07	−32.67	−4.87	−0.45	−0.01	−0.00
255.15	−574.24	−108.00	−45.56	−18.96	−8.24	−1.08	−0.30	−0.01	−0.00
260.15	−114.81	−24.04	−10.42	−4.96	−1.92	−0.35	−0.15	0.01	0.01
265.15	−24.87	−5.47	−2.52	−1.63	−0.60	0.20	0.06	0.01	0.01
270.15	−6.37	−1.77	−1.12	0.06	−0.27	−0.01	0.03	0.00	0.00
275.15	−1.29	−0.23	−0.34	−0.45	−0.08	−0.02	−0.01	−0.01	0.00
280.15	−0.41	0.25	0.23	−0.31	−0.33	−0.28	−0.03	0.00	0.00
285.15	−0.10	−0.11	0.34	0.04	−0.38	−0.00	0.02	0.00	−0.01
290.15	0.10	−0.17	0.37	0.01	0.12	−0.01	0.00	0.01	−0.01
295.15	0.18	−0.05	0.34	0.02	0.00	0.03	0.02	−0.01	0.01
300.15	−0.39	0.06	0.00	−0.01	−0.02	0.01	−0.00	−0.01	−0.01

Table SM-7.4.2. As Table SM-7.4.1, but for $200 \leq p/\mathrm{hPa} \leq 1000$ and $235.15 \leq T/K \leq 300.15$.

T/K	p/hPa								
	1000	900	800	700	600	500	400	300	200
235.15	−2348501.21	−2351652.63	−2354810.49	−2357969.59	−2361135.13	−2364304.50	−2367477.70	−2370657.33	−**2373838.17**
240.15	−180590.11	−180791.41	−180992.64	−181195.73	−181398.75	−181599.77	−181802.65	−182007.38	−182210.10
245.15	−21312.37	−21334.25	−21354.89	−21375.87	−21397.20	−21418.86	−21439.30	−21461.65	−21482.76
250.15	−3219.34	−3221.96	−3225.06	−3228.62	−3231.30	−3234.44	−3236.69	−3239.42	−3242.61
255.15	−573.79	−574.47	−575.15	−574.62	−575.31	−576.01	−576.71	−577.42	−578.13
260.15	−114.80	−115.80	−115.05	−115.90	−116.11	−115.67	−115.73	−116.26	−116.16
265.15	−25.26	−25.46	−25.37	−25.00	−25.39	−25.49	−25.30	−25.88	−25.12
270.15	−6.14	−5.65	−6.11	−6.51	−5.85	−6.13	−6.37	−6.54	−5.65
275.15	−1.05	−1.94	−1.95	−1.10	−1.33	−1.67	−1.14	−1.69	−1.37
280.15	−0.30	−0.16	−0.25	−0.57	−0.16	0.02	−0.03	−0.30	−0.81
285.15	0.32	0.04	−0.56	−0.54	0.09	−0.53	−0.54	0.06	0.35
290.15	−0.39	0.35	−0.23	−0.29	0.19	0.27	−0.05	0.16	−0.02
295.15	0.37	0.07	0.23	−0.08	0.09	−0.22	−0.05	−0.35	−0.18
300.15	0.40	0.08	0.17	−0.26	−0.28	0.12	−0.00	0.29	0.06

SM-7.5 Sound Speed

Table SM-7.5.1. Relative deviation, $(w - w_{num})\,/w_{num}$ in parts per billion (ppb), of the sound speed w using the analytically determined low-density water fraction from the sound speed w_{num} using the numerically determined low-density water fraction for $0.1 \le p/\text{MPa} \le 400$ and $253.15 \le T/\text{K} \le 303.15$.

p/MPa	T/K											
	253.15	255.65	258.15	260.65	263.15	268.15	273.15	278.15	288.15	293.15	298.15	303.15
0.1	**821.07**	404.34	201.15	101.40	51.64	14.15	3.99	0.56	0.57	−0.58	−0.80	0.31
10.0	448.92	223.04	111.30	57.55	29.48	7.95	2.86	−0.01	−0.21	−0.95	0.61	0.75
20.0	234.26	117.26	60.43	31.02	16.78	3.72	1.70	−0.38	−0.77	−0.71	0.01	−0.06
30.0	119.03	60.25	31.89	15.52	9.10	1.67	0.41	−0.19	−0.64	0.51	−0.07	−0.10
40.0	58.63	30.90	15.99	8.99	4.99	1.56	0.91	0.58	−0.99	−0.86	−0.08	−0.08
50.0	28.91	14.82	7.36	3.36	1.42	0.12	0.70	−0.97	0.25	0.05	0.10	0.01
60.0	13.87	7.19	3.63	1.70	0.60	−0.47	−0.58	−0.15	−0.00	0.02	−0.09	−0.11
70.0	5.50	2.65	2.53	1.83	1.35	−0.10	−0.62	−0.03	0.02	−0.05	−0.05	−0.05
80.0	3.58	0.52	0.98	0.13	1.48	−0.36	1.09	0.07	0.10	−0.13	−0.10	0.09
90.0	1.46	0.20	0.18	−0.68	−0.39	−0.56	−0.99	−0.01	−0.10	−0.09	−0.14	−0.00
100.0	1.06	0.02	1.31	0.31	0.24	−0.46	−0.08	0.09	0.01	0.07	−0.07	0.05
110.0	0.43	−0.39	−0.92	−1.03	0.85	0.02	−0.05	0.13	0.10	−0.08	−0.09	0.12
120.0	1.41	0.03	1.43	−0.06	−0.07	−0.06	−0.10	0.09	−0.04	0.04	0.15	0.02
130.0	−0.68	0.04	−0.05	0.02	−0.12	−0.13	−0.09	−0.08	−0.12	0.00	−0.12	0.11
140.0	0.01	0.04	−0.13	0.01	0.02	−0.11	0.11	−0.05	−0.03	0.13	0.13	0.12
150.0	−0.15	−0.03	−0.08	−0.16	0.06	−0.14	−0.02	0.14	0.15	−0.06	−0.15	−0.08
160.0	0.01	−0.05	−0.09	−0.13	−0.02	0.02	−0.05	−0.12	0.02	−0.09	0.07	0.13
170.0	−0.13	−0.13	−0.00	0.07	−0.08	0.16	0.05	−0.09	−0.17	0.09	−0.08	−0.01
180.0	0.15	−0.20	−0.14	0.06	0.11	0.14	−0.17	−0.03	0.15	0.18	−0.08	0.12

Continuation of Table SM-7.5.1.

p/MPa	T/K											
	253.15	255.65	258.15	260.65	263.15	268.15	273.15	278.15	288.15	293.15	298.15	303.15
190.0	0.15	−0.01	0.04	0.08	−0.05	0.08	−0.05	−0.01	0.03	0.15	0.10	−0.01
200.0	−0.16	0.14	−0.12	−0.16	0.14	0.10	0.11	0.10	0.20	−0.13	−0.14	−0.12
210.0	−0.07	0.16	−0.03	−0.16	0.14	−0.04	−0.03	−0.23	0.12	0.10	0.06	−0.04
220.0	−0.01	0.09	0.13	−0.03	0.24	0.09	−0.09	0.02	0.16	−0.05	0.20	−0.23
230.0	−0.15	−0.19	−0.15	−0.13	−0.17	0.20	0.01	−0.10	0.09	−0.16	−0.11	−0.02
240.0	−0.07	−0.01	0.14	0.07	−0.05	0.20	−0.12	−0.11	0.01	0.23	0.13	−0.08
250.0	0.26	−0.02	−0.00	0.21	−0.12	−0.13	0.15	−0.25	0.19	−0.09	−0.23	0.08
260.0	−0.27	−0.01	0.23	−0.04	−0.24	−0.01	−0.23	−0.03	−0.11	0.05	0.03	−0.00
270.0	0.27	−0.25	−0.09	0.06	0.17	0.17	−0.21	0.08	−0.14	0.27	−0.01	−0.00
280.0	0.07	−0.04	0.25	−0.09	−0.16	−0.04	−0.08	−0.01	−0.22	0.00	0.01	−0.00
290.0	0.16	0.15	−0.02	0.17	−0.15	0.06	−0.17	0.24	−0.01	−0.00	−0.00	−0.01
300.0	−0.26	0.31	0.15	0.14	−0.13	−0.27	−0.31	0.00	0.01	0.00	−0.00	−0.00
310.0	−0.00	−0.10	−0.10	0.27	−0.07	−0.35	0.01	0.00	−0.00	0.00	0.01	−0.01
320.0	−0.16	0.28	−0.22	−0.01	−0.01	0.01	0.00	0.01	−0.00	0.00	0.00	−0.00
330.0	0.00	−0.01	−0.01	0.01	−0.01	0.00	0.01	0.01	0.00	−0.01	−0.00	−0.00
340.0	0.01	0.00	−0.00	−0.01	0.01	−0.01	−0.01	−0.00	0.01	−0.00	−0.00	−0.00
350.0	−0.00	0.00	−0.01	0.00	0.00	0.01	−0.01	−0.01	0.00	−0.00	0.00	0.00
360.0	−0.00	−0.00	0.01	0.00	0.01	−0.00	0.01	−0.01	−0.01	−0.00	0.00	0.01
370.0	−0.00	−0.00	−0.01	0.00	−0.00	−0.01	0.00	−0.01	−0.01	0.00	−0.00	0.00
380.0	0.00	−0.00	0.01	−0.01	−0.00	0.00	0.01	−0.00	−0.01	−0.00	0.00	0.00
390.0	0.00	−0.00	0.01	−0.00	0.00	−0.01	0.01	0.00	0.00	−0.00	−0.00	−0.00
400.0	−0.00	0.00	−0.00	−0.00	−0.00	0.00	−0.01	−0.00	−0.00	−0.00	−0.00	0.00

Table SM-7.5.2. As in Table SM-7.5.1, but for $0.1 \leq p/\mathrm{MPa} \leq 1000$ and $273.15 \leq T/\mathrm{K} \leq 303.15$.

p/MPa	T/K			
	273.15	283.15	293.15	303.15
0.1	**3.99**	−0.37	−0.58	0.31
50.0	0.70	−0.92	0.05	0.01
100.0	−0.08	−0.04	0.07	0.05
150.0	−0.02	−0.00	−0.06	−0.08
200.0	0.11	0.12	−0.13	−0.12
250.0	0.15	−0.04	−0.09	0.08
300.0	−0.31	−0.01	0.00	−0.00
350.0	−0.01	0.00	−0.00	0.00
400.0	−0.01	−0.00	−0.00	0.00
450.0	0.01	0.00	−0.00	0.00
500.0	−0.00	−0.00	0.00	−0.00
550.0	−0.00	0.00	0.00	−0.00
600.0	−0.00	0.00	0.00	0.00
650.0	0.00	−0.00	0.00	0.00
700.0	−0.00	−0.00	0.00	−0.00
750.0	−0.00	0.00	0.00	−0.00
800.0	−0.00	0.00	−0.00	−0.00
850.0	0.00	0.00	−0.00	0.00
900.0	0.00	0.00	−0.00	0.00
950.0	−0.00	−0.00	0.00	−0.00
1000.0	−0.00	−0.00	0.00	0.00

SM-7.6 Gibbs Energy and Entropy

Table SM-7.6.1. Relative deviation, $(\hat{g} - \hat{g}_{\mathrm{num}})\,/\hat{g}_{\mathrm{num}}$ in units of parts per billion (ppb), of the specific Gibbs energy $\hat{g}$ using the analytically determined low-density water fraction (Section 3.2) from the specific Gibbs energy $\hat{g}_{\mathrm{num}}$ using the numerically determined low-density water fraction for $200 \leq p/\mathrm{hPa} \leq 1000$ and $235.15 \leq T/K \leq 300.15$.

T/K	p/hPa								
	1000	900	800	700	600	500	400	300	200
235.15	−945.79	−946.99	−948.19	−949.39	−950.60	−951.81	−953.02	−954.23	**−955.45**
240.15	−21.86	−21.87	−21.89	−21.90	−21.92	−21.93	−21.95	−21.96	−21.98
245.15	−0.89	−0.89	−0.89	−0.89	−0.89	−0.90	−0.90	−0.90	−0.90
250.15	−0.05	−0.05	−0.05	−0.05	−0.05	−0.05	−0.05	−0.05	−0.05
255.15	−0.00	−0.00	−0.00	−0.00	−0.00	−0.00	−0.00	−0.00	−0.00
260.15	−0.00	−0.00	−0.00	−0.00	−0.00	−0.00	−0.00	−0.00	−0.00
265.15	−0.00	−0.00	−0.00	−0.00	−0.00	−0.00	−0.00	−0.00	−0.00
270.15	0.00	0.00	0.00	−0.04	−0.00	−0.00	−0.00	0.00	−0.00
275.15	0.00	0.00	−0.00	−0.00	0.00	0.00	0.00	0.03	−0.00
280.15	0.00	−0.00	−0.00	−0.00	−0.00	0.00	−0.00	−0.00	−0.00
285.15	0.00	−0.00	−0.00	−0.00	0.00	−0.00	0.00	0.00	−0.00
290.15	−0.00	0.00	−0.00	−0.00	−0.00	−0.00	0.00	−0.00	0.00
295.15	−0.00	−0.00	−0.00	−0.00	0.00	−0.00	−0.00	0.00	0.00
300.15	−0.00	0.00	−0.00	−0.00	−0.00	0.00	−0.00	−0.00	0.00

Table SM-7.6.2. Relative deviation, $(\widehat{s}-\widehat{s}_{\mathrm{num}})/\widehat{s}_{\mathrm{num}}$ in parts per billion (ppb), of the specific entropy $\widehat{s}$ using the analytically determined low-density water fraction from the specific entropy $\widehat{s}_{\mathrm{num}}$ using the numerically determined low-density water fraction for $200 \leq p/\mathrm{hPa} \leq 1000$ and $235.15 \leq T/K \leq 300.15$.

T/K	p/hPa								
	1000	900	800	700	600	500	400	300	200
235.15	−856729.10	−857726.22	−858725.32	−859724.49	−860725.64	−861727.81	−862731.01	−863736.16	**−864741.39**
240.15	−110411.71	−110520.32	−110628.86	−110738.51	−110848.08	−110956.40	−111065.82	−111176.35	−111285.62
245.15	−19971.77	−19990.14	−20007.34	−20024.87	−20042.71	−20060.87	−20077.87	−20096.66	−20114.28
250.15	−4485.25	−4488.50	−4492.40	−4496.96	−4500.27	−4504.24	−4506.97	−4510.34	−4514.38
255.15	−1196.76	−1198.08	−1199.41	−1198.21	−1199.56	−1200.92	−1202.28	−1203.66	−1205.04
260.15	−376.89	−380.17	−377.66	−380.42	−381.08	−379.64	−379.78	−381.51	−381.14
265.15	−149.70	−150.88	−150.34	−148.10	−150.40	−150.99	−149.88	−153.30	−148.76
270.15	−105.74	−97.25	−105.11	−112.00	−100.59	−105.53	−109.49	−112.48	−97.18
275.15	29.42	54.17	54.67	30.89	37.33	46.74	31.88	47.23	38.31
280.15	2.52	1.36	2.13	4.81	1.37	−0.15	0.25	2.57	6.81
285.15	−1.66	−0.21	2.90	2.81	−0.48	2.77	2.82	−0.33	−1.82
290.15	1.48	−1.35	0.89	1.10	−0.72	−1.02	0.20	−0.62	0.08
295.15	−1.14	−0.20	−0.69	0.23	−0.26	0.65	0.15	1.06	0.54
300.15	−1.02	−0.20	−0.43	0.66	0.70	−0.32	0.00	−0.74	−0.15

1. IAPWS G12-15. Guideline on Thermodynamic Properties of Supercooled Water. Technical report, The International Association for the Properties of Water and Steam, Stockholm, Sweden, July 2015, 2015, [http://www.iapws.org].
2. Wagner, W.; Pruß, A. The IAPWS formulation 1995 for the thermodynamic properties of ordinary water substance for general and scientific use. *J. Phys. Chem. Ref. Data* **2002**, *31*, 387–535.
3. IAPWS R6-95. Revised Release on the IAPWS Formulation 1995 for the Thermodynamic Properties of Ordinary Water Substance for General and Scientific Use. Technical report, The International Association for the Properties of Water and Steam, Dresden, Germany, September 2016, 2016, [http://www.iapws.org].
4. Feistel, R.; Wagner, W. A new equation of state for H_2O ice Ih. *J. Phys. Chem. Ref. Data* **2006**, *35*, 1021–1047. doi:10.1063/1.2183324.
5. IAPWS R10-06. Revised Release on the Equation of State 2006 for H_2O Ice Ih. Technical report, The International Association for the Properties of Water and Steam, Doorwerth, The Netherlands, September 2009, 2009, [http://www.iapws.org].
6. Feistel, R. A new extended Gibbs thermodynamic potential of seawater. *Progress in Oceanography* **2003**, *58*, 43–114. doi:10.1016/S0079-6611(03)00088-0.
7. Feistel, R. A Gibbs function for seawater thermodynamics for -6 to 80 °C and salinity up to 120 g kg^{-1}. *Deep-Sea Research I* **2008**, *55*, 1639–1671. doi:10.1016/j.dsr.2008.07.004.
8. IAPWS R13-08. Release on the IAPWS Formulation 2008 for the Thermodynamic Properties of Seawater. Technical report, The International Association for the Properties of Water and Steam, Berlin, Germany, September 2008, 2008, [http://www.iapws.org].
9. Lemmon, E.W.; Jacobsen, R.T.; Penoncello, S.G.; Friend, D.G. Thermodynamic properties of air and mixtures of nitrogen, argon, and oxygen from 60 to 2000 K at pressures to 2000 MPa. *J. Phys. Chem. Ref. Data* **2000**, *29*, 331–385. doi:10.1063/1.1285884.
10. Hyland, R.W.; Wexler, A. Formulations for the thermodynamic properties of the saturated phases of H_2O from 173.15 K to 473.15 K. *Trans. Am. Soc. Heat. Refrig. Air Cond. Eng.* **1983**, *89*, 500–519.
11. Harvey, A.H.; Huang, P.H. First-principles calculation of the air–water second virial coefficient. *Int. J. Thermophys.* **2007**, *28*, 556–565. doi:10.1007/s10765-007-0197-8.
12. Feistel, R.; Wright, D.G.; H.-J. Kretzschmar.; Hagen, E.; Herrmann, S.; Span, R. Thermodynamic properties of sea air. *Ocean Sci.* **2010**, *6*, 91–141.
13. Ostwald, W. Studien über die Bildung und Umwandlung fester Körper. 1. Abhandlung: Übersättigung und Überkaltung. *Zeitschrift für Physikalische Chemie* **1897**, *22*, 289–330. doi:https://doi.org/10.1515/zpch-1897-2233.
14. Schmelzer, J.; Möller, J.; Gutzow, I. Ostwald's rule of stages: the effect of elastic strains and external pressure. *Z. Phys. Chemie* **1998**, *204*, 171–181.
15. Schmelzer, J.W.P.; Schmelzer Jr., J.; Gutzow, I.S. Reconciling Gibbs and van der Waals: a new approach to nucleation theory. *J. Chem. Phys.* **2000**, *112*, 3820–3831, [https://doi.org/10.1063/1.481595].
16. Schmelzer, J.W.P. Kinetic and thermodynamic theories of nucleation. *Mater. Phys. Mech.* **2003**, *6*, 21–33.
17. Schmelzer, J.W.P.; Fokin, V.M.; Abyzov, A.S.; Zanotto, E.D. How do crystals form and grow in glass-forming liquids: Ostwald's rule of stages and beyond. *Int. J. Appl. Glass Sci.* **2010**, *1*, 16–26, [https://doi.org/10.1111/j.2041-1294.2010.00003.x].
18. Gutzow, I.S.; Schmelzer, J.W.P. Growth of Clusters and of Ensembles of Clusters: Ostwald Ripening and Ostwald's Rule of Stages. In *The Vitreous State*; Gutzow, I.S.; Schmelzer, J.W.P., Eds.; Springer, Berlin, Heidelberg, 2013; pp. 367–393.
19. Schmelzer, J.W.P.; Abyzov, A.S. How Do Crystals Nucleate and Grow: Ostwald's Rule of Stages and Beyond. In *Thermal Physics and Thermal Analysis. Hot Topics in Thermal Analysis and Calorimetry, vol 11*; Šesták, J.; Hubík, P.; Mareš, J., Eds.; Springer: Cham, 2017.
20. Hedges, L.O.; Whitelam, S. Limit of validity of Ostwald's rule of stages in a statistical mechanical model of crystallization. *The Journal of Chemical Physics* **2011**, *135*, 164902, [https://doi.org/10.1063/1.3655358]. doi:10.1063/1.3655358.

21. Schmelzer, J.W.P.; Boltachev, G.S.; Baidakov, V.G. Classical and generalized Gibbs' approaches and the work of critical cluster formation in nucleation theory. *J. Chem. Phys.* **2006**, *124*, 194503, [https://doi.org/10.1063/1.2196412].
22. Schmelzer, J.W.P.; Boltachev, G.S.; Baidakov, V.G. Is Gibbs' thermodynamic theory of heterogeneous systems really perfect? In *Nucleation Theory and Applications*; Schmelzer, J.W.P., Ed.; Wiley-VCH, Berlin-Weinheim, 2005; pp. 418–446.
23. Gibbs, J.W. On the equilibrium of heterogeneous substances. *Trans. Connecticut Acad. Arts and Sci.* **1877**, *III*, 44–520.
24. Debenedetti, P.G.; Stanley, H.E. Supercooled and glassy water. *Physics Today* **2003**, pp. 40–46.
25. Hellmuth, O.; Schmelzer, J.; Feistel, R. Ice-crystal nucleation in water: thermodynamic driving force and surface tension. Part I: theoretical foundation. *Entropy* **2020**, *22*, 50. doi:10.3390/e22010050.
26. Abyzov, A.S.; Fokin, V.M.; Yuritsyn, N.S.; Rodrigues, A.M.; Schmelzer, J.W.P. The effect of heterogeneous structure of glass-forming liquids on crystal nucleation. *Journal of Non-Crystalline Solids* **2017**, *462*, 32 – 40, [https://doi.org/10.1016/j.jnoncrysol.2017.02.004].
27. Feistel, R. Report of the IAPWS/TPWS Task Group "Correlation Equations for the Melting Curve and the Sublimation Curve". Technical report, The International Association for the Properties of Water and Steam, Draft December 13, 2006, unpublished material, 2006.
28. IAPWS. Release on the IAPWS Formulation 1995 for the Thermodynamic Properties of Ordinary Water Substance for General and Scientific Use. Technical report, The International Association for the Properties of Water and Steam, Fredericia, Denmark, September 1996, 1996, [http://www.iapws.org].
29. IAPWS. Release on an Equation of State for H_2O Ice Ih. Technical report, The International Association for the Properties of Water and Steam, Witney, UK, September 2006, 2006, [http://www.iapws.org].
30. Gellert, W.; Küstner, H.; Hellwich, M.; Kästner (eds.), H. *Kleine Enzyklopädie Mathematik*; VEB Bibliographisches Institut, Leipzig, 1979.
31. Press, W.H.; Teukolsky, S.A.; Vetterling, W.T.; Flannery, B.P. *Numerical Recipes in Fortran 77. The Art of Scientific Computing. Second Edition. Volume 1 of Fortran Numerical Recipes*; Cambridge University Press, New York, 1996.

List of Tables

Table SM-3.1.1 Parameter values for the equation of state given by Equations (SM-3.1)–(SM-3.8). Taken from IAPWS G12-15 [1] (Table 1 therein). 6

Table SM-3.1.2 Parameter values for the dimensionless regular background Gibbs energy ψ^{r} in Equation (SM-3.3). Taken from IAPWS G12-15 [1] (Table 2 therein). 6

Table SM-3.2.1 Derivatives of $L(\tau,\pi)$ and $\psi^{\mathrm{r}}(\tau,\pi)$. To simplify the annotation of the derivatives of ψ^{r} (right column), the following shorthand definitions are used: $\overline{\tau}=\tau+1$ and $\overline{\pi}=\pi+\pi_0$. Taken from IAPWS G12-15 [1] (Table 3 therein). 7

Table SM-3.3.1 Regression coefficients a_{k}, n_{k} for $p_{\mathrm{M}}(T)$, Equation (SM-3.17), and b_{k} for the $T_{\mathrm{M}}(p)$, Equation (SM-3.18). Taken from Feistel [27]. 9

Table SM-3.4.1 Subintervals for the numerical determination of the equilibrium mole fraction of low-density water, x_{e}, in dependence on $L(\tau,\pi)$ and $\omega(\pi)$. The “min” function returns the smallest value of its arguments. Taken from IAPWS G12-15 [1] (Table 4 therein). 9

Table SM-3.5.1 Thermodynamic reference values for check of the correct computer implementation. Taken from IAPWS G12-15 [1] (Table 5 therein). 11

Table SM-5.1 Deviations of the numerically determined values of mass density $\widehat{\varrho}$, thermal expansion coefficient α_p, isothermal compressibility κ_T, isobaric heat capacity c_p, speed of sound w, equilibrium mole fraction of low-density water, x_{e}, and the ordering field L using the root finder of Press *et al.* [31] (Section 9.1 therein) from the IAPWS G12-15 [1] reference values (subscript $\star$, Table SM-3.5.1). Integer n denotes the number of equally spaced segments of the root interval of x_{e}. Relative deviations are given in parts per billion (ppb). Bold-styled values denote extrema. 15

Table SM-6.1 Deviation of the analytically determined mass density $\widehat{\varrho}$, thermal expansion coefficient α_p, isothermal compressibility κ_T, isobaric heat capacity c_p, speed of sound w, equilibrium mole fraction of low-density water, x_{e}, and the ordering field L according to Section SM-3.1 from the IAPWS G12-15 [1] reference values (subscript $\star$, Table SM-3.5.1). Relative deviations are given in parts per billion (ppb). Bold-styled values denote extrema. 17

Table SM-7.1.1 Relative deviation, $(\widehat{\varrho}-\widehat{\varrho}_{\mathrm{num}})/\widehat{\varrho}_{\mathrm{num}}$ in parts per billion (ppb), of the mass density $\widehat{\varrho}$ using the analytically determined low-density water fraction from the mass density $\widehat{\varrho}_{\mathrm{num}}$ using the numerically determined low-density water fraction at p=0.101325 MPa. 18

Table SM-7.1.2 As Table SM-7.1.1, but for $0.1 \le p/\mathrm{MPa} \le 400$ and $253.15 \le T/\mathrm{K} \le 303.15$. 19

Table SM-7.1.3 As Table SM-7.1.1, but for $0.1 \le p/\mathrm{MPa} \le 1000$ and $235.15 \le T/\mathrm{K} \le 303.15$. 23

Table SM-7.1.4 As Table SM-7.1.1, but for $200 \le p/\mathrm{hPa} \le 1000$ and $235.15 \le T/K \le 300.15$. 24

Table SM-7.2.1 Deviation, $(\alpha_p-\alpha_{p,\mathrm{num}})\,/\,(10^{-4}\,\mathrm{K}^{-1})$, of the thermal expansivity α_p using the analytically determined low-density water fraction from the thermal expansivity $\alpha_{p,\mathrm{num}}$ using the numerically determined low-density water fraction for $0.1 \le p/\mathrm{MPa} \le 600$ and $245.5 \le T/\mathrm{K} \le 288.0$. 26

Table SM-7.3.1 Relative deviation, $(\kappa_T-\kappa_{T,\mathrm{num}})/\kappa_{T,\mathrm{num}}$ in parts per billion (ppb), of the isothermal compressibility κ_T using the analytically determined low-density water fraction from the thermal expansivity $\kappa_{T,\mathrm{num}}$ using the numerically determined low-density water fraction for $0.1 \le p/\mathrm{MPa} \le 190$ and $235.15 \le T/\mathrm{K} \le 300.15$. 28

Table SM-7.4.1 Relative deviation, $(\widehat{c}_p - \widehat{c}_{p,\mathrm{num}}) / \widehat{c}_{p,\mathrm{num}}$ in parts per billion (ppb), of the isobaric heat capacity $\widehat{c}_p$ using the analytically determined low-density water fraction from the isobaric heat capacity $\widehat{c}_{p,\mathrm{num}}$ using the numerically determined low-density water fraction for $0.1 \leq p/\mathrm{MPa} \leq 190$ and $235.15 \leq T/\mathrm{K} \leq 300.15$. 30
Table SM-7.4.2 As Table SM-7.4.1, but for $200 \leq p/\mathrm{hPa} \leq 1000$ and $235.15 \leq T/K \leq 300.15$. 30
Table SM-7.5.1 Relative deviation, $(w - w_{\mathrm{num}}) / w_{\mathrm{num}}$ in parts per billion (ppb), of the sound speed w using the analytically determined low-density water fraction from the sound speed w_{num} using the numerically determined low-density water fraction for $0.1 \leq p/\mathrm{MPa} \leq 400$ and $253.15 \leq T/\mathrm{K} \leq 303.15$. 31
Table SM-7.5.2 As in Table SM-7.5.1, but for $0.1 \leq p/\mathrm{MPa} \leq 1000$ and $273.15 \leq T/\mathrm{K} \leq 303.15$. 33
Table SM-7.6.1 Relative deviation, $(\widehat{g} - \widehat{g}_{\mathrm{num}}) / \widehat{g}_{\mathrm{num}}$ in units of parts per billion (ppb), of the specific Gibbs energy $\widehat{g}$ using the analytically determined low-density water fraction (Section 3.2) from the specific Gibbs energy $\widehat{g}_{\mathrm{num}}$ using the numerically determined low-density water fraction for $200 \leq p/\mathrm{hPa} \leq 1000$ and $235.15 \leq T/K \leq 300.15$. 34
Table SM-7.6.2 Relative deviation, $(\widehat{s} - \widehat{s}_{\mathrm{num}}) / \widehat{s}_{\mathrm{num}}$ in parts per billion (ppb), of the specific entropy $\widehat{s}$ using the analytically determined low-density water fraction from the specific entropy $\widehat{s}_{\mathrm{num}}$ using the numerically determined low-density water fraction for $200 \leq p/\mathrm{hPa} \leq 1000$ and $235.15 \leq T/K \leq 300.15$. 35

© 2020 by the authors. Submitted to *Journal Not Specified* for possible open access publication under the terms and conditions of the Creative Commons Attribution (CC BY) license (http://creativecommons.org/licenses/by/4.0/).
